# Microbial Biodegradation of Plastics: Methods for Assessing Degradation Mechanisms and Optimization Strategies

**DOI:** 10.3390/microorganisms14102335

**Published:** 2026-10-13

**Authors:** Yuming Qiao, Yunqiao Wang, Guoqiang Han, Jingzhen Zhang, Zhiyuan Yao, Bin He, Pengfei Hu, Li Jiang

**Affiliations:** 1 Institute of One Health Science, School of Civil Engineering and Future Cities, Ningbo University, Ningbo 315211, China; 2 International Science and Technology Cooperation Base for Coastal Agricultural Water Resilience and One Health of Zhejiang Province, Ningbo 315211, China; 3 Sichuan WEIPU Testing Technology Co., Ltd., Chengdu 610100, China

**Keywords:** plastic, microbial degradation, enzyme, mechanism, efficiency, challenges

## Abstract

With the rapid growth of plastic production, plastic pollution has become increasingly widespread and persistent in the environment. This persistent pollution poses potential risks to ecosystems and human health. Microbial biodegradation offers a promising approach to plastic waste management because it operates under mild reaction conditions and has potential for sustainable application. This review summarizes recent advances in the major microbial groups involved in plastic biodegradation, methods for assessing degradation efficiency, underlying mechanisms, and strategies for improving degradation performance. Assessment methods are discussed, from physicochemical characterization to biochemical validation. Meanwhile, it emphasizes that real plastic biodegradation and degradation efficiency require multiple complementary lines of evidence to judge in a more complete and objective way. The complete process and molecular mechanisms of microbial plastic degradation are then described, including microbial attachment and biofilm formation, enzymatic degradation, intracellular metabolism, and mineralization of degradation products. In addition, it summarizes various efficiency optimization strategies for degradation, including plastic pretreatment, bioaugmentation, and regulation of environmental parameters. The lack of standardized assessment, limited practical translation, and ecological risks are the identified key challenges. Finally, standardized efficiency evaluation strategies, plastic upcycling, and emerging technologies for subsequent development were discussed. This review aims to provide a reference for understanding microbial plastic degradation.

## 1. Introduction

Plastics have become important materials in modern society because of their low cost, stable properties, and broad applications. However, rapidly expanding production, environmental persistence, and inadequate recycling have contributed to severe plastic pollution [1]. Poorly managed plastic waste can fragment into microplastics and nanoplastics through photo-oxidation, mechanical abrasion, and biological processes in the environment [2]. These particles are widely distributed in marine, freshwater, soil, and agricultural systems, where they may inhibit plant growth and cause genotoxicity, disturb animal communities in aquatic sediments, and affect fundamental biogeochemical processes such as carbon and nitrogen cycles [3,4,5]. Therefore, the management and effective treatment of plastic waste have become important issues in environmental pollution control.

Plastic waste management should be chosen based on polymer properties and waste stream characteristics. Mechanical recycling remains a preferred route for relatively clean, single-polymer waste streams, where established sorting, washing, and re-extrusion processes can recover materials for reuse, although their properties may deteriorate during repeated processing [6,7]. Pyrolysis, on the other hand, plays an irreplaceable role in processing complex and contaminated waste streams that are unsuitable for mechanical recycling, including end-of-life tires, single-use medical plastics, multilayer packaging, and fiber-reinforced composites, from which it recovers oil, gas, and solid char feedstocks [8,9,10]. Biological treatment of plastic acts as a complementary disposal pathway. Biological degradation exhibits practical value mainly for biodegradable polymers, as well as for highly dispersed plastic residues which are technically difficult to collect and recycle [6,8,11]. Biodegradation of plastic is relatively mild, low cost, and can be coupled with resource recovery, making it an important technology for plastic waste management. Microorganisms can colonize plastic surfaces and use polymers or their degradation products as carbon sources during plastic transformation [12,13].

Numerous previous studies have devoted efforts toward mining functional microorganisms capable of degrading synthetic plastics. To date, plastic-degrading microbial resources involve bacteria, fungi, and algae, offering abundant biological candidates for developing plastic biodegradation technologies. Typical microbial genera, including *Pseudomonas*, *Bacillus*, *Rhodococcus*, *Aspergillus*, and *Rhodotorula*, have been widely identified [14,15]. These microbes can degrade common plastic products such as polyethylene (PE), polyethylene terephthalate (PET), polypropylene (PP), and polyvinyl chloride (PVC) [15,16,17]. Physicochemical characterization methods, including scanning electron microscopy (SEM), Fourier transform infrared spectroscopy (FTIR), and gravimetric weight loss measurement, are widely adopted to monitor material structural alterations during degradation [18]. Biochemical methods, such as stable isotope tracing [13], enzyme activity assays [19], and CO_2_ release monitoring [20] are also applied to evaluate whether genuine biodegradation proceeds. Nevertheless, existing evaluation frameworks still suffer notable imperfections that only apply isolated detection indices and fail to construct multi-dimensional evidence chains for rigorous confirmation of authentic microbial mineralization, potentially overestimating real degradation performance. In addition, to overcome the low intrinsic degradation capacity of wild-type microbes and promote practical translation, multiple improvement strategies have been explored, including physical and chemical pretreatment [21,22], immobilized systems [23], enzyme engineering [24], synthetic biology [22], bioaugmentation [25], and regulation of environmental parameters [26]. Despite the substantial progress achieved in the accumulation of microbial resources and degradation performance improvement, several critical unsolved bottlenecks, such as lack of standardized assessment, leakage of microplastics and secondary pollutants, and ecological risks associated with microorganisms, still restrict the practical application and large-scale industrialization of plastic microbial degradation technology.

Compared with existing review articles on microbial plastic biodegradation, the present review provides several distinct contributions and novelties. First, polymer-specific multi-dimensional assessment criteria to distinguish superficial surface damage from genuine polymer mineralization were emphasized, highlighting the risk of overinterpretation merely relying on weight loss or morphological observation. Second, the molecular degradation mechanisms, performance optimization strategies, and potential ecological risks were integrated, providing systematic references for advancing standardized evaluation and real-world translation of plastic biodegradation technologies. Third, this review dissects core bottlenecks restricting practical plastic biodegradation. It critically analyzes a set of long-underappreciated limiting factors, such as physical constraints imposed by polymer crystallinity, mass transfer barriers at the enzyme-plastic interface, and the substantial performance gap between laboratory-scale observations and real-world application scenarios.

This review addresses three key questions: (1) Which microorganisms have demonstrated plastic-degrading potential, and how does their degradation performance vary among different plastics; (2) How is microbial plastic biodegradation currently assessed, and what biological processes and molecular mechanisms underlie plastic degradation; (3) What strategies have been developed to enhance microbial plastic biodegradation efficiency. To answer the above questions, this review systematically summarizes polymer structural features governing degradability, major microbial groups participating in plastic biodegradation, multi-level plastic biodegradation assessment methods, complete biodegradation mechanisms, performance-enhancing approaches, bottlenecks, and future perspectives. Details of the literature search and screening strategy are provided in the Supplementary Materials (Figure S1, Table S1, and Text S1). Collectively, this review can offer theoretical references for multi-dimensional experimental evaluation setups, biodegradation efficiency enhancement, as well as risk assessment for plastic bioremediation. Meanwhile, it is expected to inspire future research toward bridging lab-scale observations and real plastic waste disposal, and provide supporting insights for formulating standardized assessment specifications and developing practical biological plastic recycling strategies.

## 2. Classification and Structural Characteristics of Plastic Polymers

The chemical structural features of plastics exert a substantial influence on their microbial degradability [18,27,28]. Typical plastic materials can be broadly divided into traditional recalcitrant synthetic plastics and artificially developed biodegradable polymers, which exhibit distinct structural properties and microbial degradation susceptibility [18,27]. Conventional recalcitrant synthetic plastics mainly include PE, PP, polystyrene (PS), and PVC, which possess chemically stable carbon-carbon (C-C) backbones [27,28]. The inert C-C bonds cannot be directly cleaved by hydrolases and require preliminary oxidative modification prior to microbial utilization [18,29,30]. Degradation of PS is hindered by stable aromatic side chains, while PVC degradation is further complicated by an essential dechlorination process during depolymerization [31]. Therefore, conventional recalcitrant synthetic plastics show strong inherent recalcitrance toward biological degradation [18,27,32,33]. In contrast, ester-containing polymers, including PET, polylactic acid (PLA), polycaprolactone (PCL), and polyester-based polyurethane (PU), contain hydrolysable ester linkages that can be cleaved under appropriate enzymatic or environmental conditions [32,33,34]. Although PET is a petroleum-derived polymer, its ester linkages make it susceptible to hydrolytic attack by esterase-type enzymes [34,35,36]. As a typical polyether, polyethylene glycol (PEG) possesses ether linkages along its backbone. PEG is highly hydrophilic owing to ether oxygen atoms and terminal hydroxyl groups. The degradability of PEG is strongly dependent on molecular weight size [37].

In the solid state, most plastics exist as either semicrystalline or amorphous solid state materials. Representative semicrystalline polymers include PET, PE, high-density polyethylene (HDPE), low-density polyethylene (LDPE), linear low-density polyethylene (LLDPE), PP, PCL and PLA, whereas typical amorphous polymers are PS and PVC with molecular chains hardly fold into ordered crystalline structures. In semicrystalline plastics, enzymatic degradation driven by microorganisms occurs predominantly in disordered amorphous regions with high chain accessibility, while densely packed crystalline domains are barely susceptible to erosion [38,39]. Densely packed crystalline domains impose a major physical barrier, which prevents enzymes from penetrating into the interior of crystal lattices, resulting in drastically reduced degradation rates [38,40]. The overall degradability of a given semicrystalline polymer is strongly influenced by its crystallinity, thermal history, and pretreatment. The activity of plastic-degrading enzymes in highly crystalline regions is significantly weakened, which has become the main obstacle to achieving complete depolymerization [38]. It should be noted that an amorphous microstructure does not inherently confer better enzymatic degradability. Although PS and PVC lack tightly packed crystalline domains and thus present fewer physical barriers for enzyme access, their chemically inert C-C backbones with pendant aromatic rings for PS and chlorine substituents for PVC develop strong chemical recalcitrance. Consequently, PS and PVC are generally more resistant to enzymatic depolymerization than semicrystalline polyesters such as PLA, PCL, and PET, even though these polyesters are limited by crystallinity-driven physical limitations. Beyond primary backbone chemistry and crystalline morphology, multiple material properties including molecular weight, additives, surface hydrophobicity, polymer morphology, and polymer thermal history also jointly govern microbial adhesion, enzyme substrate interaction, and substrate accessibility, thereby determining the overall biodegradation performance of different plastic materials [18,27,38,41,42]. Distinguishing polymer classes provides a structural basis for understanding why different microorganisms, enzymes, assessment methods, and pretreatment strategies are required for different plastics.

## 3. Core Microbial Groups Involved in Plastic Biodegradation

### 3.1. Bacteria

Bacteria of the genera *Pseudomonas*, *Bacillus*, and *Rhodococcus* are the main microorganisms involved in plastic biodegradation (Table 1) [14]. *Pseudomonas* grows rapidly and has diverse metabolic pathways, showing degradation capacity for different plastics such as PET and PE [16]. As a key PET-degrading bacterium, the Gram-negative bacterium *Pseudomonas putida* can cause a 22.86% weight loss of PET within 30 days [43]. It secretes functional esterase enzymes to attack ester linkages of PET, producing mono(2-hydroxyethyl) terephthalate (MHET), bis(2-hydroxyethyl) terephthalate (BHET), and terephthalic acid, and possesses complete intracellular metabolic pathways for further utilizing these PET-derived hydrolysis products [43]. *Pseudomonas* sp. SH5B and *Pseudomonas aeruginosa* SH6B achieved a PE degradation rate of 25% in 120 days [44]. *Bacillus* species can form spores that are resistant to adverse environments and survive for long periods of time under nutrient deficiency or significant temperature fluctuations. The *Bacillus cereus* NJD1 strain screened from plastic waste dumps exhibited prominent LDPE biodegradation capability, with a 43% weight loss of LDPE films observed after 120 days of cultivation [45]. Likewise, *Bacillus subtilis* AP-04 screened in the intestines of plastic-fed mealworms achieved a weight loss of 36.55% for 60 days [46]. In addition, *Bacillus cereus* SHBF2 caused a weight loss of 6.77% for PP and 6.87% for PE during a 60-day experiment [17]. The genus *Rhodococcus* possesses a remarkable capacity for utilizing hydrophobic compounds. *Rhodococcus pyridinivorans* reduced the weight of LDPE by 5.2% within 24 h [47]. *Rhodococcus erythropolis* also showed obvious degradation of LDPE [48].

In addition to these three major genera, many other bacteria can degrade plastics (Table 1), with differences in degradation efficiency depending on the polymer type. For example, *Paenibacillus* sp. are able to degrade multiple plastics and can degrade 6% of PP within 20 days [53], while *Paenibacillus lautus* degraded 3.73% of PS after 60 days [52]. *Acinetobacter baumannii* similarly displayed broad substrate specificity, degrading 33.3% of PP and 27.1% of PVC within 50 days [54]. Other bacteria appear more specialized. *Paenarthrobacter nitroguajacolicus* effectively degraded LDPE [48], whereas two *Proteus mirabilis* strains isolated from oil-contaminated soil reduced LDPE film weight by 71.80% and 89.72%, respectively, after 78 days [57]. *Aeromonas* degraded 5% of PP within 20 days [53], while *Neobacillus* sp. PVCKKU3 degraded 7.5% of PVC after 35 days [55]. *Brevibacillus parabrevis* CGK45 degraded 4.03% of HDPE within 90 days [59]. Closely related microorganisms often share physiological traits and metabolic functions. Phylogenetic comparisons with characterized plastic-degrading bacteria may therefore facilitate the identification of additional microorganisms with degradation potential.

### 3.2. Fungi

Fungi are ubiquitous across diverse ecosystems and represent an important microbial group for investigating plastic biodegradation [28,65]. Various fungal genera, such as *Aspergillus*, *Coriolopsis,* and *Fusarium*, are known to degrade plastics (Table 1). Among them, the degradation of plastics by *Aspergillus* is the most widely studied. Within 60 days, *Aspergillus niger* and *Aspergillus flavus* degraded PVC by 25.3% and 23.6%, respectively [15]. *Aspergillus glaucus* achieved a 32% degradation rate of PVC in only 28 days under different culture conditions. In addition, *Aspergillus glaucus* and *Aspergillus niger* degraded PE by up to 25% and 40% within 28 days [62].

Yeasts also exhibit potential for plastic depolymerization in microbial-mediated plastic-degrading systems. Several yeast genera, including *Moesziomyces*, *Rhodotorula*, and *Candida*, with plastic-degrading activities mainly target hydrolysable polyester substrates including poly(butylene adipate-co-terephthalate) (PBAT), poly(butylene succinate-co-adipate) (PBSA), PCL, and PET. The native degrading representative, *Moesziomyces antarcticus* (formerly *Pseudozyma antarctica*), secretes the extracellular enzyme PaE for efficient degradation of biodegradable polyesters such as PBAT and PBSA, while it shows limited activity toward PET and PE [71]. *Cryptococcus flavus* GB-1 produces the degrading enzyme CfCLE and forms clear hydrolysis zones on emulsified PBSA plates, while its enzymatic activity can be further improved via UV mutagenesis [70]. *Yarrowia lipolytica* does not possess strong plastic-degrading capability, yet it serves as an attractive metabolic engineering chassis for heterologous expression of PETase, cutinase, or Lip2 lipase for PET and PCL biodegradation [73,74]. *Rhodotorula mucilaginosa* from marine degraded UV-pretreated ^13^C-labelled PE, and both ^13^CO_2_ evolution and assimilation of PE-derived carbon into yeast biomass were detected, corresponding to a degradation rate of 3.8%yr^−1^ [63]. Nevertheless, this performance relies on UV pretreatment and cannot be extrapolated directly to untreated PE waste under real-world conditions. *Candida tropicalis* (recently renamed *Yamadazyma tropicalis*) exhibits degradation capacity toward LDPE by secreting extracellular oxidative enzymes [12]. Marine isolate *Meyerozyma carpophila* M6.0.2 is another candidate PE-degrading yeast, although its underlying degradation mechanism remains poorly characterized [75]. In summary, yeast-driven biodegradation of polyesters has obtained substantial research progress [51]. For polyethylene (PE/LDPE) with stable C-C backbones, experimental evidence of yeast-mediated degradation remains scarce.

Except for *Aspergillus* and yeasts, *Coriolopsis gallica* degraded 19% of PVC within 60 days [15]. The *Fusarium solani* separated from landfills reduced the weight of LDPE by 20.83% after 45 days [65]. *Schizophyllum commune* and *Lentinus sajor-caju* from the same environment were able to degrade LLDPE [64]. Micromycetes, a group of microscopic fungi, have also demonstrated the ability to degrade different plastics. *Penicillium corylophilum* CNMN-FP-04 caused a 6.06% reduction in the weight of LDPE after 30 days [66]. The soil micromycete *Fusarium oxysporum* resulted in a 22% weight loss of PET after 30 days when PET was used as the sole carbon source [69]. *Phanerochaete chrysosporium* degraded 7% of PS within 35 days, and *Phellinus noxius* degraded 16% of expanded polystyrene (EPS) within 30 days [61]. However, most of these newly isolated fungi still exhibit limited plastic degradation rates under non-optimized, environmentally relevant conditions [42,76], and their performance frequently deteriorates when transferred from laboratory media to real-world waste matrices.

### 3.3. Algae and Algae-Bacteria Consortium

In addition to bacteria and fungi, some algae can also directly participate in plastic biodegradation, thus expanding the microbial resources for plastic bioremediation (Table 1). *Uronema africanum* Borge induced surface erosion and structural deterioration of LDPE within 30 days [72]. Under light conditions, Algae can also promote the collaborative degradation of plastics by microorganisms through building a local microenvironment. In algae–bacteria consortia, plastic deformation, functional-group changes, and degradation potential have been observed alongside the release of dissolved organic matter by *Desmodesmus* sp. under different plastic stresses [77]. However, the regulatory mechanisms of different algae species, plastic types, and environmental conditions still need to be further studied and clarified. In addition, algae have also been used to construct composite engineered degradation platforms. After the engineered plastic-degrading enzyme complex was immobilized on the surface of *Chlorella vulgaris*, the PET biodegradation efficiency increased four times compared with the free enzyme. The immobilization of the enzyme also promoted algae biomass accumulation and chlorophyll production, showing the degradation and resource recovery potential of the algae enzyme composite system [23].

### 3.4. Microbial Community

Microbial communities can outperform individual strains through functional interactions, metabolic complementation, and ecological succession [78,79]. Different strains perform distinct degradation steps, including oxidation, hydrolysis, and small-molecule metabolism [79,80]. *Streptomyces* sp. initiates plastic oxidation and provides attachment sites for *Arthrobacter* sp., which subsequently degrades intermediates and enhances PE degradation [78]. With a mixed culture of *Delftia acidovorans* and *Bacillus* sp., the biodegradation efficiency of LDPE and PS was higher than that of pure culture [25]. The synthetic microbial communities of *Bacillus licheniformis*, *Bacillus subtilis,* and *Paenibacillus xylanilyticus* can also increase the weight loss of LDPE [81]. Nevertheless, competition for substrates and inhibitory metabolic interactions among strains can reduce overall degradation efficiency. The collaboration between *Stenotrophomonas maltophilia* and *Bacillus velezensis* can improve the PS biodegradation efficiency, whereas the addition of *Acinetobacter radioresistens* reduced the biodegradation efficiency [50]. The microbial community formed on plastics also has the potential for plastic degradation. Different plastics can enrich different functional bacterial groups, and niche succession of the microbial community was related to environmental conditions, such as soil properties, plastic exposure history, pollution duration, and pollution intensity [82]. Therefore, plastic biodegradation is essentially an ecological process involving division of labor, collaboration, and dynamic succession among multiple bacterial groups.

## 4. Methods for Assessing Biodegradation Efficiency

### 4.1. Physicochemical Characterization

**Figure 1:**
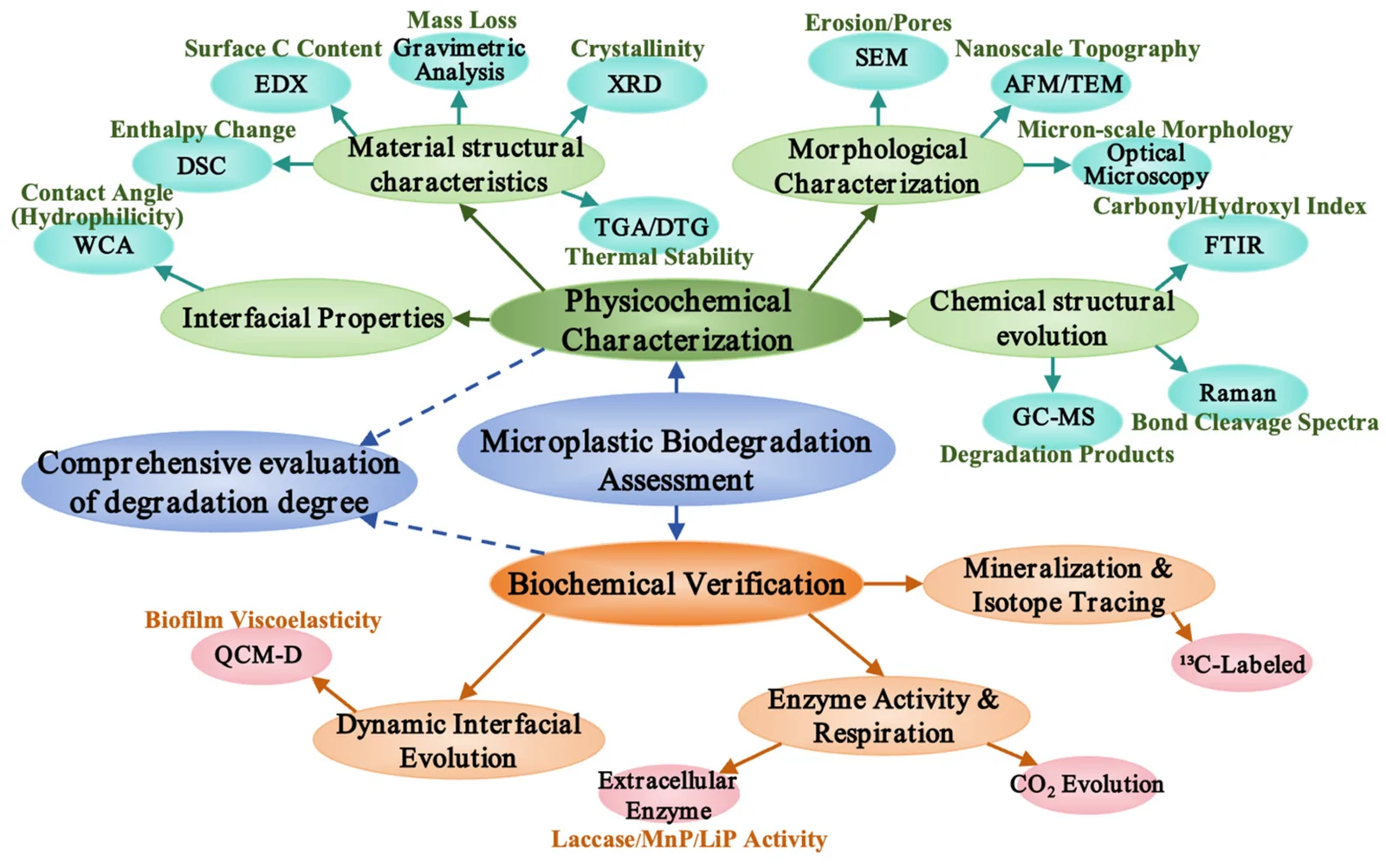
The methods for assessing plastic biodegradation based on physicochemical characterization and biochemical validation. Green and cyan represent physicochemical characterization, orange and pink represent biochemical verification, and blue represents the overall assessment framework. Solid arrows show the relationships among components, whereas dashed arrows indicate that the two assessment approaches need to be integrated for a comprehensive evaluation.

The verification of plastic biodegradation relies on the application of multidimensional physicochemical characterization (Figure 1), which covers morphological characterization, material structure characteristics, chemical structure evolution, and interfacial properties. For morphological characterization, microscopic imaging can reveal surface changes of plastics, such as erosion, holes, and cracks [57,83]. Atomic force microscopy (AFM), transmission electron microscopy (TEM), and SEM can further provide nanoscale or complementary surface information [61,66,72].

At the level of material structure parameters, the most basic method is gravimetric analysis, which can simply and intuitively estimate material weight loss during biodegradation through the degradation rate [45]. X-ray diffraction (XRD) can reflect changes in crystallinity and phase composition [12]. And energy-dispersive X-ray spectroscopy (EDX), commonly coupled with SEM, can reveal changes in surface elemental composition and provide auxiliary evidence of plastic deterioration [57]. Differential scanning calorimetry (DSC) is used to measure the energy absorbed or released by plastics during heating or cooling to determine whether decomposition of the polymer structure has occurred [84]. Thermogravimetric analysis (TGA) and derivative thermogravimetric analysis (DTG) characterize changes in material thermal stability, thereby reflecting the occurrence of plastic degradation [54,77]. These test methods can characterize the degree of microbial damage to polymer structure from the perspective of material-property degradation.

At the chemical structure level, FTIR is used to analyze changes in surface functional groups during plastic degradation. The formation of carbonyl, hydroxyl, and other oxygen-containing functional groups reflects oxidation and scission of polymer chains [17,59,85]. FTIR has also been used to calculate semi-quantitative parameters, including hydrophilicity enhancement, the component ratio of carbonyl, and crystallinity change [86]. Gas chromatography-mass spectrometry (GC-MS) can qualitatively and quantitatively identify new compounds generated during plastic degradation [15]. Raman spectroscopy can detect changes in characteristic molecular vibrations associated with chemical-structure alteration during plastic degradation [54].

At the level of interfacial property characterization, water contact angle measurement can reflect changes in wettability and hydrophilicity on the plastic surface. Because polar functional groups increase during plastic degradation, the surface of plastic usually shows a decrease in water contact angle, which can serve as auxiliary evidence of surface oxidation and changes in interfacial properties [54].

### 4.2. Biochemical Validation

Biochemical validation of plastic degradation commonly examines mineralization, enzyme activity, and interfacial dynamics (Figure 1). Stable isotope tracing using ^13^C-labeled plastics directly verifies the mineralization of plastic carbon by tracking its conversion into ^13^CO_2_ or microbial biomass [13]. In freshwater and river sediment systems, ^13^C-labeled PE was mineralized by indigenous microbiota. Nevertheless, the observed degradation rates were extremely low, suggesting that complete degradation may require hundreds to thousands of years [13,87].

Enzyme activity and respiration measurements provide real-time evidence for plastic degradation. When *Neopestalotiopsis phangngaensis* degraded LDPE, extracellular enzymes were detected, such as laccase, manganese peroxidase, and lignin peroxidase [19]. When *Achromobacter xylosoxidans* degraded PE and nylon, oxidases related to C-C bond cleavage, including laccase and peroxidase, were also released [56]. Respiration measurements can quantify CO2 evolution associated with biodegradation, while isotope-labelled substrates can more directly establish its plastic-derived origin [13,20].

In addition, quartz crystal microbalance dissipative technology (QCM-D) provides a surface-sensitive approach for real-time characterization of microbial–plastic interfacial processes. QCM-D is based on the piezoelectric oscillation of a quartz crystal and simultaneously records changes in resonance frequency (Δf) and energy dissipation (ΔD). Changes in Δf mainly reflect variations in the acoustically coupled mass at the sensor surface, whereas ΔD provides information on the rigidity and viscoelastic properties of the attached layer, thereby allowing microbial attachment, biofilm growth, and structural changes at the interface to be monitored dynamically [85,88]. For example, QCM-D with polystyrene-coated sensors has been used to continuously monitor bacterial attachment and biofilm development, where frequency changes reflect biomass accumulation and dissipation changes indicate variations in the viscoelastic properties of the attached biofilm [85]. It has also been applied to quantify the deposition and release of polystyrene microplastics on *Pseudomonas fluorescens* biofilms, demonstrating its potential for investigating dynamic interactions between plastics and microbial biofilms [89].

### 4.3. Selection of Assessment Methods

Drawing a clear conceptual boundary between superficial surface erosion/weight loss and true polymer mineralization is critical. Surface cracking, pitting, and gravimetric reduction can arise not only from microbial depolymerization, but also from abiotic oxidation, additive leaching, or partial oligomer release [2,90]. Physicochemical characterization can intuitively reflect changes in plastic surface morphology, polymer structure, and material properties, but it is difficult to distinguish the real degradation process [18,27]. Relying solely on physicochemical characterization may overestimate biodegradation performance [90]. For example, increases in carbonyl and hydroxyl groups may also result from abiotic oxidation induced by heating, light exposure, or metal-ion catalysis [2,91]. Strict abiotic blank control groups are essential for all plastic biodegradation experiments [90]. Specifically, sterile systems without microorganisms are established under identical incubation conditions, including culture medium composition, temperature, incubation duration, light conditions, and metal ion concentration. Any functional group changes, weight loss, or structural damage observed in the abiotic control group are defined as background abiotic oxidation and excluded from microbial degradation evaluation. Moreover, evidence that plastic-derived carbon is ultimately converted into CO_2_, H_2_O, or microbial biomass is also required [92]. Biochemical validation can directly reflect the transformation and mineralization of plastic carbon through CO_2_ measurement, isotope tracing, and changes in enzyme activity, but it provides limited information on changes in polymer structure [18,27,92]. Accordingly, integrating physicochemical characterization with biochemical validation enables a more comprehensive assessment of the actual biodegradation process and its efficiency [20].

Furthermore, the selection of assessment methods should also depend on polymer chemistry. More concretely, for ester-bond-containing polymers such as PET and PU, besides routine physicochemical characterization, quantitative measurement of characteristic hydrolysis products, such as terephthalic acid (TPA), MHET, and BHET for PET, is required, together with mineralization monitoring to verify real biodegradation [39]. For C-C backbone polymers such as PE and PP, key observations include molecular weight reduction and oxidative spectral signals from FTIR or Raman, which must be cross-checked against mineralization evidence, like CO_2_ evolution or isotope tracing, since carbonyl group elevation can also originate from abiotic oxidation [93,94]. For aromatic polystyrene (PS and EPS), detection of aromatic intermediate metabolites (styrene oligomers, aromatic ketones or alcohols) should serve as important auxiliary evidence alongside mineralization assays [95]. For PVC, valid assessment should combine structural degradation observation with quantitative chloride ion release data, as dechlorination represents a key step of PVC biodegradation [96]. Therefore, standardized assessment should establish polymer-specific evidence chains rather than applying the same analytical protocol to all plastics [90].

## 5. Biodegradation Processes and Molecular Mechanisms

### 5.1. Microbial Attachment and Biofilm Formation on Plastic

**Figure 2:**
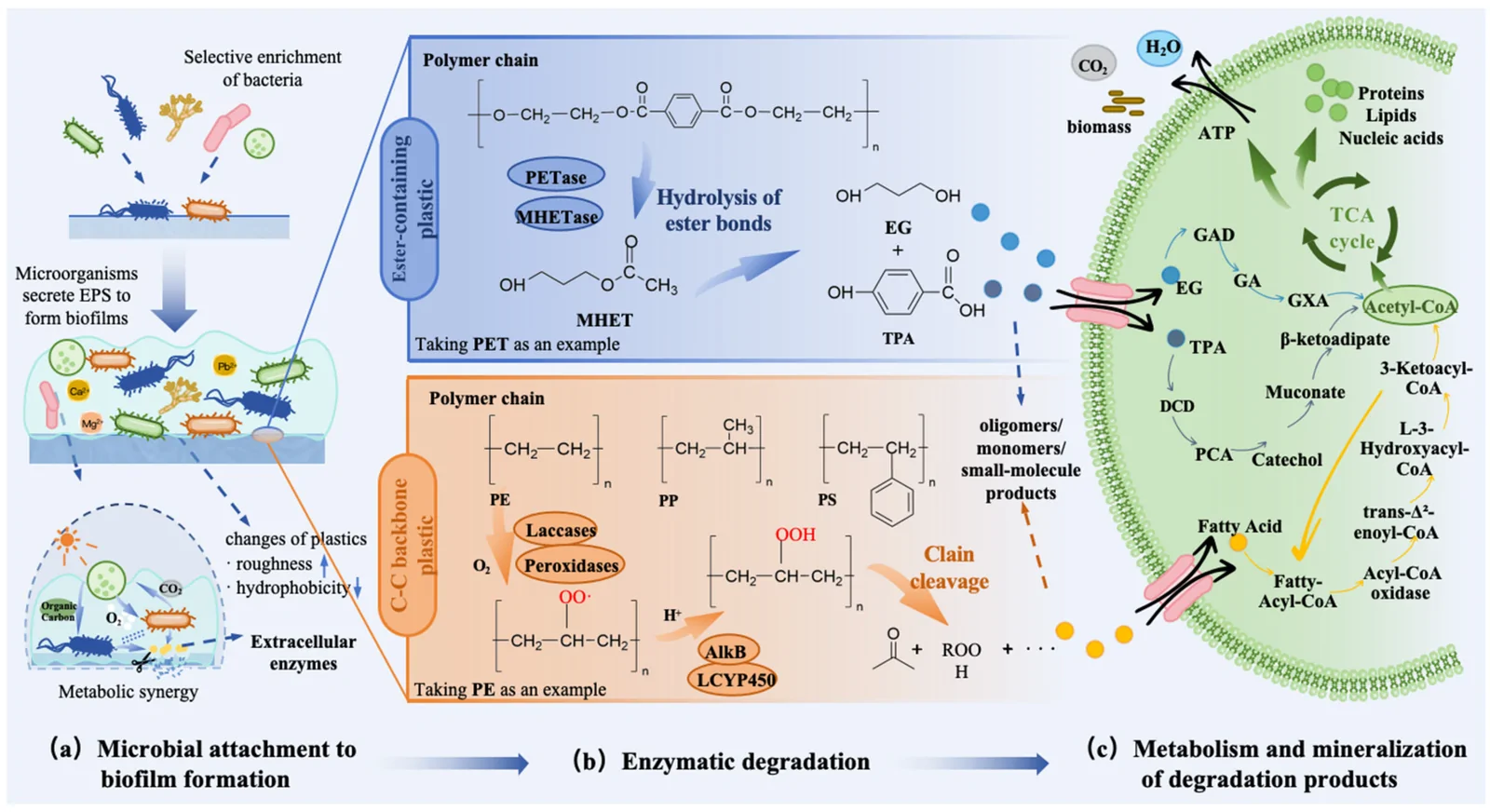
Microbial biodegradation mechanisms of plastics. (**a**) Microorganisms attach to plastic surfaces and secrete extracellular polymeric substances to form biofilms. (**b**) Enzymatic degradation processes of ester-bond-containing polymers represented by PET and C-C-backbone polymers represented by PE. (**c**) Oligomers, monomers, and small-molecule products are transported into cells and converted through central metabolic pathways, leading to biomass formation, ATP generation, and mineralization to CO_2_ and H_2_O. Arrows indicate the progression of biodegradation and metabolic pathways, while the graphical symbols schematically represent microorganisms, enzymes, degradation products, and cellular components; the ellipses indicate additional intermediate products that are not individually shown.

Plastic biodegradation usually begins with microbial attachment and colonization on plastic surfaces (Figure 2), which is influenced by the surface properties of microorganisms and plastic substrates. The capacity of bacteria to colonize plastic surfaces is therefore a critical determinant of initial attachment. Despite belonging to the same genus, *Bacillus amyloliquefaciens* BSM-2 exhibits stronger colonization ability on inert LDPE films than strain BSM-1 [60]. Early colonization is selective and may transiently enrich microorganisms capable of hydrocarbon degradation. For instance, obligate hydrocarbon-degrading bacteria (OHCB) can account for 5.8% of early colonizers on PE surfaces in marine environments, yet their relative abundance declines as the biofilm matures [97]. Plastic-associated microbial communities subsequently undergo dynamic succession from initial attachment to mature biofilm formation. Because polymers differ in their physicochemical properties, they can support distinct microbial community structures across environments, time points, and stages of biofilm development [98].

Biofilms on plastic are composed of microorganisms, extracellular polymeric substances secreted by microorganisms, and extracellular DNA. The protein components of extracellular polymeric substances not only affect the structural stability of the biofilm, but also participate in dynamic regulation of biofilm. Trypsin can significantly reduce the biofilm of the PE-degrading bacterium *Rhodococcus ruber* C208 to a single-layer structure, while proteinase K promotes the formation of a more stable multilayer biofilm [99]. The proteins in extracellular polymeric substances may affect the ability of microorganisms to colonize plastic surfaces by regulating cell adhesion and biofilm maturation [99]. In addition, biofilm development is also regulated by cell-to-cell communication systems, such as quorum sensing (QS) [100]. In Gram-negative bacteria, this regulation is commonly mediated by N-acyl homoserine lactone (AHL), whereas Gram-positive bacteria frequently employ peptide-based signaling [100]. As microbial cell density increases on plastic surfaces, the accumulation of QS signaling molecules can activate QS-regulated genes involved in cell adhesion, extracellular polymeric substance production, and motility [101]. Through these regulatory networks, QS can coordinate the transition from initial surface colonization to structured biofilm development [101]. With the improvement of biofilm structural maturity, QS signals also regulate the expression levels of key degrading enzymes (laccase, peroxidase, hydrolase), directly linking biofilm development with plastic biodegradation activity [101,102]. The mature biofilms increase surface roughness and maintain close contact between microbial cells and hydrophobic polymer surfaces and can concentrate extracellular enzymes at the interface of plastic and biofilm, thereby facilitating subsequent polymer degradation [102,103]. Moreover, QS regulation also mediates microbial community synergism and niche succession on plastic surfaces, coordinating the metabolic division of labor among different functional strains [104], which might optimize the overall biodegradation performance of microbial consortia. The combination of *Arthrobacter* sp., growing in suspension, and *Streptomyces* sp., capable of forming abundant biofilms on PE, results in stronger degradation than either strain alone [78].

### 5.2. Enzymatic Degradation of Plastic

Enzymatic degradation of plastic mainly proceeds via two core reaction pathways. Polymers with ester bonds are hydrolyzed, whereas polymers with C-C backbones are oxidized or hydroxylated. The key enzymes responsible for these processes mainly include hydrolases, together with different families of oxidoreductases represented by laccases, peroxidases, and alkane hydroxylases (Figure 2).

Hydrolases mainly act on plastics containing ester bonds, such as PET and PU. By catalyzing the hydrolysis of ester bonds, these enzymes decompose polymers into monomers that can be absorbed and utilized [32,68]. As the predominant hydrolases for enzymatic plastic degradation, esterases (typified by cutinases and lipases) belong to the serine hydrolase superfamily [35,105]. At the catalytic level, serine hydrolases possess a conserved Ser-His-Asp catalytic triad forming a metal-free catalytic pocket. The histidine and aspartate residues activate the nucleophilic serine residue, which directly attacks ester bonds of polymer substrates [35]. The reaction is a water-dependent hydrolytic cleavage, breaking ester-bond-containing polymers (e.g., PET, PU) into oligomers and monomers without generating free radicals. PETase from *Ideonella sakaiensis* is a specific cutinase that converts PET into MHET. Then, MHETase hydrolyzes MHET into TPA and ethylene glycol (EG) [34,106]. The lipase-producing strain *Penicillium simplicissimum* has also been verified to depolymerize PET, yielding MHET and TPA as major degradation products [68].

In contrast, PE and PP possess stable, non-hydrolysable C–C backbones and generally require oxidative activation before chain scission can occur [18,27]. For PE, oxidation mainly introduces hydroxyl and carbonyl groups along the relatively unsubstituted hydrocarbon chain, thereby increasing surface polarity and facilitating subsequent chain cleavage [18,27,29]. For PP, pendant methyl groups create tertiary carbon sites that are relatively more susceptible to oxidative attack, although crystallinity and structural complexity may still restrict degradation [18,27]. These oxidative processes can involve laccases, peroxidases, and alkane hydroxylases, depending on the microorganism and polymer [18,27]. Laccases and laccase-like multicopper oxidases are copper-dependent oxidases built around multicopper active site clusters [107]. They belong to the multicopper oxidase superfamily with conserved copper-binding motifs, and copper ions constitute their indispensable catalytic cofactor. Relying on electron transfer mediated by copper ions between substrates and molecular oxygen, these enzymes generate reactive radical intermediates via single-electron oxidation [107]. This radical-forming microenvironment enables oxidative modification of inert C-C backbones in polyethylene, introducing polar carbonyl and hydroxyl groups, with molecular oxygen serving as the terminal electron acceptor and being reduced to water [29,107]. It is noteworthy that some oxidative enzymes for plastic depolymerization, including manganese peroxidase and lignin peroxidase, are heme-iron-dependent peroxidases [108]. For example, *Penicillium simplicissimum* can produce laccase and manganese peroxidase (MnP) [67]. Treatment of PE with crude laccase and manganese peroxidase preparations from *Penicillium simplicissimum* resulted in measurable weight loss and changes in oxygen-containing functional groups, supporting the involvement of these enzymes in the oxidative degradation of PE [67]. AlkB is a potential hydrocarbon-chain oxidase. C-H bonds on polymer side chains can be hydroxylated by AlkB, which increases the oxidative reactivity of polymer segments and facilitates subsequent chain cleavage and molecular weight decline [30].

Notably, plastic enzymatic degradation depends on the sequential and synergistic effects of multiple enzymes. During PU degradation by *Alicycliphilus denitrificans* BQ1, extracellular esterase and carbamate-cleaving enzymes act sequentially and cooperatively to depolymerize polymer substrates [32]. Similarly, two marine *Marinobacter* sp. isolates and one *Bacillus subtilis* isolate were shown to degrade LDPE, with esterase production implicated in polymer modification and the release of fatty-acid degradation products [58]. Initially, alkane hydroxylase, laccase, and peroxidase co-mediate the oxidation of C-H bonds, introducing hydroxyl and carbonyl groups and triggering C-C backbone cleavage. Auxiliary hydrolases (e.g., esterases) subsequently convert the oxidized intermediates into products with lower molecular weights products [58]. The overall apparent degradation rate is governed by the slowest step among these sequential processes.

As microplastics are insoluble solid substrates, their enzymatic degradation represents a typical interfacial catalysis process constrained by multiple sequential mass transfer reaction steps [36,109]. These interfacial constraints include diffusion resistance of enzymes toward hydrophobic polymer surfaces, dynamic adsorption and desorption equilibrium between enzymes and solid substrates, and product inhibition caused by accumulating oligomeric intermediates [40,107]. First, enzymes in aqueous solution must overcome external diffusion resistance across the liquid boundary layer to reach the solid plastic surface. Once arriving at the surface, enzymes require favorable orientation for effective adsorption; either too weak or excessive adsorption will reduce local catalytic performance [110]. Even after surface adsorption of enzymes, polymer chain accessibility is further governed by polymer packing density and chain mobility, where densely packed crystalline domains hinder delivery of polymer segments toward enzyme active sites. Moreover, timely release of monomeric and oligomeric degradation intermediates from the solid-liquid interface into the aqueous phase constitutes an easily overlooked backward mass transfer step. Local accumulation of degradation products may trigger product inhibition effects [111].

Microbial biofilms exert dual influences on mass transfer. Biofilm colonization enriches extracellular enzymes at the interface to elevate local enzyme concentration, yet dense biofilm matrices may introduce additional diffusion barriers [112]. Variations in stirring intensity, particle size assays, and the Damköhler number can help diagnose mass transfer limitation phenomena [113]. Consequently, merely increasing total enzyme activity or enzyme dosage in bulk solution does not necessarily produce proportional improvements in degradation performance. Future optimization strategies should simultaneously consider external mass transfer conditions, enzyme-substrate interfacial adsorption behavior, polymer chain accessibility, and removal of degradation intermediates [114]. Systematically comparable mass transfer parameters remain scarce for diverse polymer types, particle sizes, aged materials, and real environmental matrices, which restricts the establishment of universal optimal operating conditions.

### 5.3. Metabolism and Mineralization of Plastic Degradation Intermediates

The metabolism of plastic degradation intermediates mainly occurs intracellularly (Figure 2). Microorganisms catabolize small molecular products released from enzymatic depolymerization to sustain energy metabolism and growth. The *Stenotrophomonas indicatrix* isolated from plastic-eating insects carries a gene cluster encoding styrene monooxygenase (StyAB), along with downstream metabolic enzyme systems, such as styrene oxide isomerase and benzaldehyde dehydrogenase. Together, these enzymes form a complete cascade that converts styrene monomers into central cellular metabolites [49]. Although PS and PEG have different chemical structures, both need to undergo terminal oxidative depolymerization before entering subsequent metabolic processes. PEG undergoes terminal hydroxyl oxidation followed by ether-bond cleavage, generating low-molecular-weight products such as EG [37]. Furthermore, the complete metabolism of plastic-derived intermediates often requires synergistic interactions among members of bacterial consortia. For example, enzymes secreted by the synergistic community of *Pseudomonas* and *Bacillus* can convert the hydrolysis intermediate BHET of PET into the monomers TPA and EG [115]. Importantly, plastic-related metabolic intermediates are not entirely eco-friendly.

Small molecules released during microbial plastic degradation are ultimately assimilated into biomass or completely mineralized to CO_2_ and H_2_O, while the associated metabolism supplies cellular energy [13,18]. After cellular uptake, TPA is initially converted into protocatechuic acid (PCA) by the terephthalate dioxygenase complex. PCA subsequently undergoes aromatic ring cleavage via the beta-ketoadipate pathway and is eventually converted into acetyl-CoA [116]. In the same way, small molecules such as EG and fatty acids are metabolized to acetyl-CoA. Acetyl-CoA further enters the tricarboxylic acid cycle to support microbial biomass synthesis or achieve thorough mineralization into CO_2_ and H_2_O [18].

## 6. Strategies for Improving Biodegradation Efficiency of Plastic

### 6.1. Pretreatment Technologies

**Figure 3:**
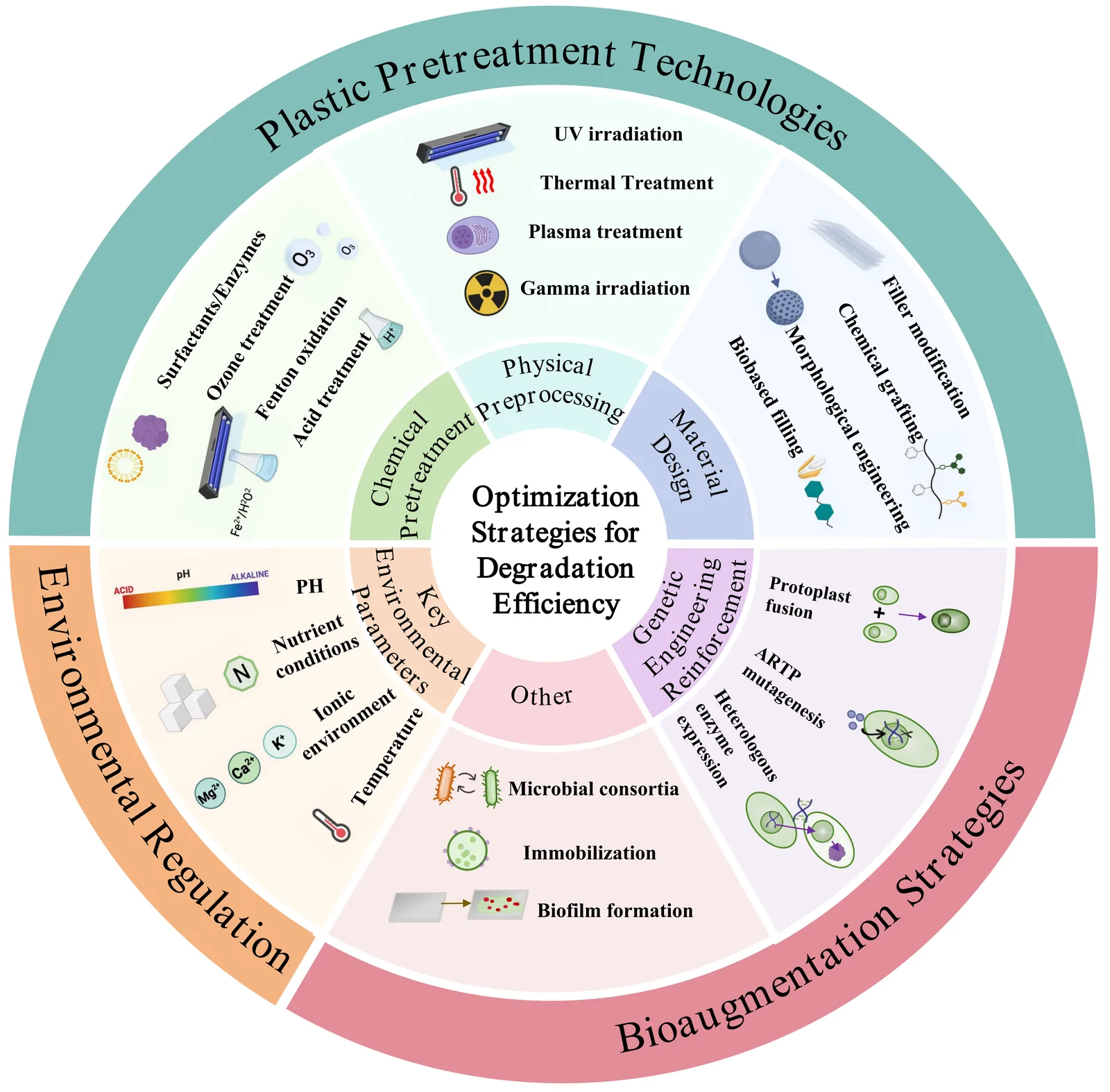
Strategies for improving plastic biodegradation efficiency.

The inherent properties of plastics, including high molecular weight, pronounced surface hydrophobicity, and high chemical stability, hinder direct microbial degradation. Pretreatment technologies can overcome these barriers by decreasing polymer molecular weight, introducing polar functional groups, and increasing surface roughness, thereby promoting microbial colonization and subsequent enzymatic degradation. According to their operating mechanisms, current pretreatment strategies can be broadly classified into physical pretreatment, chemical pretreatment, and material optimization (Figure 3).

Physical pretreatment is a fundamental approach to enhance the biodegradability of recalcitrant plastics, with prevalent techniques including ultraviolet (UV) irradiation, thermal treatment, plasma treatment, and gamma irradiation. As a convenient and efficient pretreatment method based on energy input, UV irradiation delivers exogenous energy to induce polymer chain scission and surface oxidation. This process reduces molecular weight, increases amorphous regions, and introduces oxygen-containing functional groups such as carbonyl and carboxyl groups, thereby substantially improving the bio-accessibility of plastic substrates [21]. Consistent with these structural improvements, UV-pretreated LDPE exhibits higher weight loss during composting biodegradation [117]. UV-modified PE can be more efficiently mineralized by the marine fungus *Parengyodontium album* than pristine PE [118]. Thermal treatment can also reduce the molecular weight and elongation of polyethylene films by triggering pro-oxidative chain scission. This pretreatment promotes subsequent bacterial biodegradation [119]. Gamma irradiation improves PP degradation by disrupting the polymer structure and facilitating subsequent fungal colonization [120] Beyond the direct pretreatment of polymer substrates, low-temperature plasma can also enhance LDPE biodegradation by activating the LDPE-degrading strain *Bacillus safensis* BS-10L, thereby increasing its viability, colonization, and oxidative activity [121].

Chemical pretreatment modifies polymer chain structure mainly through oxidation reactions and functional group introduction. Representative methods include Fenton oxidation, ozone treatment, acid treatment, surfactant treatment, and enzymatic pretreatment. A combination of Fenton oxidation with UVC irradiation can increase the total oxidation index and degradation of PE and PP [22]. Ozone pretreatment reduces molecular weight, generates carbonyl groups on the surface, and enhances fungal mineralization [92]. Mild acid treatment can improve the biodegradability of PE, PET, PP, and PS by promoting the formation of C=C, C=O, and O-H groups. For instance, pretreatment of PS films with 1 N nitric acid and sulfuric acid for 10 days increased bacterial degradation-induced weight loss from 2.7% to 8.3% [122].

In contrast to harsh chemical treatments, surfactant and enzymatic pretreatments represent eco-friendly alternatives. A broad range of surfactants (anionic, cationic, nonionic, and biosurfactants) and oxidoreductase/hydrolase enzymes (laccase, peroxidase, and esterase) have been validated for plastic modification. Biosurfactants are particularly important for hydrophobic polymers because their amphiphilic structure reduces surface and interfacial tension and improves the wettability, emulsification, and apparent bioavailability of polymer surfaces [123]. Surfactants can promote plastic biodegradation through several substrate- and surfactant-dependent mechanisms. For PET, suitable surfactants can reduce the interfacial tension between PET and water and improve enzyme adsorption on the polymer surface, thereby enhancing enzymatic hydrolysis [118]. Second, an aqueous micellar two-phase system composed of nonionic surfactants and polysaccharides was shown to further enhance PET depolymerization by improving contact between enzymes and substrates and continuously removing inhibitory hydrolysis products [124]. For example, surfactin produced by *Bacillus subtilis* enhanced PE biodegradation, whereas the effect of rhamnolipids has been shown to depend on polymer type and degradation conditions [125,126]. Specifically, anionic surfactant pretreatment resulted in an approximately 9% reduction in LDPE molecular weight [74]. Nevertheless, the biodegradation enhancement by biosurfactants varies considerably with polymer type, substrate crystallinity, and experimental conditions [126,127]. High production cost and difficulty in large-scale preparation remain major obstacles restricting practical field-scale deployment of biosurfactant-assisted plastic biodegradation [128].

Material design serves as another effective strategy to improve the biodegradability of conventional recalcitrant plastics. Blending biodegradable substrates, such as starch, into conventional plastics (e.g., LDPE and PP) can alleviate polymer inertness and facilitate microbial attack [129,130]. Advanced modification approaches further incorporate nanofiller incorporation, chemical grafting, and morphological optimization to regulate plastic degradability. For example, organically modified montmorillonite nanocomposites expand the interlayer spacing of composite materials and increase water permeability. Such structural optimization promotes microbial colonization within the polymer matrix and ultimately enhances the biodegradation of LDPE, PLA, and their derived composites [131]. Chemical grafting of sugar molecules onto functionalized PS introduces readily biodegradable carbohydrate moieties that increase the susceptibility of the modified polymer to fungal degradation, further improving bio-utilization efficiency [132]. At the morphological level, smaller size, higher porosity, and larger specific surface area collectively enhance substrate bioavailability and degradation performance. Porous LDPE is degraded more readily than non-porous LDPE [133].

### 6.2. Bioaugmentation Strategies

Bioaugmentation provides a powerful approach to strengthen plastic biodegradation efficiency via microbial genetic modification and strain optimization (Figure 3). Protoplast fusion serves as a strategy for strain improvement that combines the plastic-degrading traits of different parental strains, generating fusants with enhanced capabilities for plastic degradation [134]. Targeted mutagenesis technologies such as Atmospheric and Room Temperature Plasma (ARTP) represent another effective tool to break the biodegradation performance bottleneck of wild-type microbial strains. For instance, the mutant strain XZ-60S exhibited a 53.65% increase in PE biodegradation efficiency relative to the wild-type strain, which was attributed to the upregulated expression of functional genes related to laccase [135]. In addition, a more targeted strategy is to heterologously express specific and efficient plastic-degrading enzymes in optimized microbial chassis. This engineered whole-cell catalyst exhibits high degradation performance for PET [136]. Microbial synergism enables metabolic cross-feeding, in which metabolic intermediates produced by one strain can be utilized by partner microbes to support their growth and reproduction, thereby driving collaborative plastic degradation [79]. The co-culture of *Bacillus* sp. AS3 and *Sphingobacterium* sp. AS8 achieves a higher weight loss rate for LDPE than individual monocultures [137]. Nevertheless, the degrading capacity of microbial consortia is highly dependent on strain proportions and interspecific interactions. Complex co-culture systems do not always outperform the most efficient single strain, as interspecific competition among community members may inhibit microbial growth and ultimately reduce overall biodegradation efficiency [50,138].

Meanwhile, immobilization and biofilm construction can mitigate the drawbacks of free enzymes or microorganisms, which are easy to lose, susceptible to environmental impact, and difficult to maintain high interfacial activity on hydrophobic plastic surfaces. Bacteria capable of forming biofilms can maintain high local concentrations of plastic-degrading enzymes at the plastic interface and exhibit enhanced physiological adaptation and environmental persistence, thereby promoting polymer degradation [139,140].

### 6.3. Regulation of Environmental Parameters

The biodegradation of plastics is regulated by a variety of environmental factors (Figure 3). Temperature, pH, ionic conditions, and nutrient availability jointly regulate microbial degradation potential and mineralization efficiency. Temperature primarily affects plastic biodegradation by altering microbial enzyme activity. Moderate increases in temperature generally promote enzymatic reactions and microbial metabolism. For instance, the degradation rate of PE was significantly increased at 43 °C [141], while some PVC-degrading fungi exhibit optimal growth at 40 °C or 30 °C [142]. However, biodegradation does not continuously improve with rising temperature. At 30 °C, the degradation rate of biodegradable mulch residues reached 88.9%, whereas complete inhibition occurs when the temperature rises to 45 °C [26]. Thermophilic bacteria, such as *Thermus*, *Bacillus,* and *Geobacillus*, can accelerate PS degradation under high-temperature conditions of 70 °C [143]. These observations demonstrate divergent temperature responses among different microbial strains.

The pH and ionic conditions represent another group of critical environmental parameters governing plastic biodegradation. Plastic-degrading microorganisms exhibit species- and strain-specific optimal pH ranges. For example, *P. chrysosporium* achieved the maximum biodegradation rate of PE at pH 4.5 [144], while the PE-degrading fungal strain *Aspergillus flavus* displayed biodegradation activity at pH 6.5 [145]. The mangrove-derived bacteria generally achieved more efficient PP degradation under weakly acidic conditions [146]. In addition to hydrogen ion concentration, the exogenous supply of appropriate metal ions can further enhance biodegradation performance. Mg^2+^ and K^+^ can enhance PE or PP degradation by *Pseudomonas*, *Bacillus*, and *Acinetobacter* strains, whereas Ca^2+^ supplementation can facilitate LDPE degradation by *Serratia*, *Bacillus*, and *Stenotrophomonas* communities. However, high salt concentrations can suppress plastic biodegradation [146].

Nutrient limitation or over-supply fundamentally affects microbial plastic degradation. Appropriate supplementation with nutrients such as glucose and calcium can promote the growth and LDPE-degrading activity of bacteria isolated from caves [147]. By contrast, excessive nutrient inputs, particularly nitrogen sources, can inhibit biodegradable mulch-film degradation and reduce soil bacterial abundance and enzyme activity [148]. In addition, UV-C radiation can directly enhance the activity of PE- or PS-related proteases in *Pseudomonas aeruginosa* [149]. The addition of humic acid as a biostimulant facilitated biofilm formation on PE surfaces and accelerated the early phase of biodegradation [150]. Therefore, the biodegradation efficiency of plastics is not determined by one individual environmental factor, but is jointly affected by multiple environmental factors.

## 7. Challenges of Plastic Biodegradation

### 7.1. Lack of Standardized Assessment

Plastic biodegradation research still lacks a unified and complete standard for degradation efficiency assessment. Experimental conditions for verification, polymer types, pretreatment methods, and degradation-rate calculations remain non-standardized. These discrepancies make plastic degradation efficiencies difficult to compare across studies. Weight loss, surface cracking, and morphological erosion are frequently adopted to evaluate plastic biodegradation. Accordingly, gravimetric and microscopic morphological assessments alone commonly lead to overestimation of biodegradation efficiency, while intermediate accumulation may be neglected. A rigorous assessment system should cover physicochemical characterization, oligomer and monomer release, microbial assimilation, and final mineralization to verify real mineralization of plastic polymers. The lack of standardized protocols also affects strain screening, enzyme preparation evaluation, material certification, and policy governance.

### 7.2. Barriers of Practical Application

Although many methods improve plastic biodegradation efficiency under laboratory conditions, their practical application within real waste treatment systems remains challenging. Firstly, inherent plastic structure limits biotransformation. Polyolefins such as PE and PP lack easily hydrolyzable bonds. Their high molecular weight, high crystallinity, and strong hydrophobicity restrict interfacial contact with microorganisms and extracellular enzymes. Therefore, complete degradation of polyolefins usually requires long time scales [27,41]. Secondly, efficient plastic-degrading strains isolated under laboratory conditions may fail to maintain their degradative activity in real plastic waste treatment systems because of intense niche and nutrient competition with indigenous microorganisms [151]. Laboratory selection is normally performed under optimized conditions with sufficient nutrients, with single-polymer substrates, which hardly mimic complex field environments. Real waste matrices contain mixed plastic fractions, toxic additives, fluctuating physicochemical parameters, and severe constraints on interfacial mass transfer. Variable environmental conditions, complex waste composition, and mass transfer limitations can further restrict biodegradation [42]. Furthermore, genetically modified or domestically selected strains may lose key degradation-related functional traits under non-selective environmental pressure. Collectively, these factors lead to disappointing results in real plastic waste treatment systems despite promising laboratory-scale results.

### 7.3. Leakage of Microplastics and Secondary Pollutants

Biofilm-driven surface erosion, enzyme-mediated chain scission, and mechanical disturbance during incubation collectively contribute to particle size reduction of plastic. Plastic biodegradation may generate smaller micro- and nanoplastics before final degradation. Micro/nanoplastics possess high specific surface area, which enhances their capacity to adsorb heavy metals and organic pollutants in surrounding environments. Nanoplastics in particular can cross biological barriers, enter cells and tissues, and produce adverse biological effects. Besides particle fragmentation, plastic-associated additives including plasticizers, flame retardants, and pigments may be simultaneously leached out alongside polymer degradation. Many of these released additives exhibit ecotoxicity, which may impose combined toxic pressure together with micro/nanoplastic particles. Moreover, incomplete biodegradation produces various oligomeric and monomeric intermediates, some of which are more toxic or more recalcitrant than parent plastic materials. For example, a two-stage microbial treatment of PET microplastics achieved a degradation efficiency of 28% after 60 days, but the resulting degradation products reduced human endothelial cell viability by approximately 40% [152]. A subsequent detoxification step using another bacterial consortium further decreased the toxicity of these products, indicating that polymer degradation does not necessarily correspond to immediate detoxification [152]. However, this evidence demonstrates the potential toxicity of mixed products generated under controlled biodegradation conditions rather than the ecological toxicity of individual PET-derived intermediates. The compounds primarily responsible for the observed toxicity and whether comparable exposure levels occur in natural environments remain unclear. Similarly, cell-free culture supernatants generated during bacterial LDPE biodegradation showed acute toxicity toward *Daphnia magna*, with toxicity degrees ranging from 47% to 73% depending on the bacterial treatment [153]. However, because the toxic components in the culture supernatants were not individually identified, the observed effects cannot be attributed unequivocally to specific LDPE-derived intermediates rather than microbial metabolites or other components of the degradation system. Some depolymerized metabolites of PEG and polyethoxylates are potentially toxic and more difficult to degrade than the parent compounds [154].

### 7.4. Ecological Risks Associated with Microorganisms

The environmental release of exogenous degrading bacteria and engineered microorganisms raises potential biosafety concerns, including horizontal gene transfer, strain escape or persistence, unintended evolutionary changes, and disturbance of indigenous microbial communities. However, direct field evidence demonstrating these outcomes specifically in plastic biodegradation systems remains limited. Furthermore, degradation-generated micro/nanoplastics may influence horizontal gene transfer among environmental microorganisms. In a controlled conjugation experiment, secondary nanoplastics released after 60 days of PLA degradation significantly promoted antibiotic resistance gene transfer between bacteria, with the highest transfer frequency being approximately 28 times that of the control [155]. This enhancement was associated with changes in bacterial membrane permeability and the expression of conjugation-related genes [155]. However, this experiment demonstrated the promotion of plasmid-mediated antibiotic resistance gene transfer between model bacteria rather than the direct transfer of plastic degradation genes from introduced or engineered plastic-degrading microorganisms to indigenous microbial communities. Therefore, direct experimental evidence for degradation gene escape during environmental plastic biodegradation remains limited. Therefore, any practical biodegradation solution should not only pursue high degradation efficiency, but also systematically evaluate micro/nanoplastic yield, additive leakage, intermediate toxicity, and biosafety risks of introduced microorganisms.

## 8. Perspectives of Plastic Biodegradation

### 8.1. Standardization and Policy

To address the lack of standardized assessment, a unified evaluation system for plastic biodegradation should be established. Material properties, experimental conditions, control settings, and degradation rate calculations should be standardized. Assessment of degradation efficiency should shift from single indicators such as weight loss, SEM, and FTIR to evidence chains specific to each polymer, from structural damage to final mineralization. The core indicators should be selected according to polymer chemistry, including hydrolysis products for ester-containing polymers, oxidation and molecular weight changes for C-C backbone polymers, aromatic intermediates for PS, and dechlorination for PVC. This shift would improve comparability and reliability across studies. Furthermore, the environmental adaptability, stable operation, and economic feasibility need standardized evaluation under realistic application environmental conditions. To address ecological risks, comprehensive assessment systems should assess toxicity, utilization potential, and environmental risks of degradation intermediates. Control and emission policies for functional microorganisms, including engineered bacteria, should be established. Additionally, future research should pay greater attention to those controversial bottlenecks: distinguishing true mineralization from superficial material damage, overcoming accessibility limits caused by crystallinity and interfaces, and bridging the large performance gap between laboratory isolates and practical waste treatment environments.

### 8.2. Emerging Technologies

Enzyme engineering is a central strategy for enhancing plastic biodegradation through improvements in enzyme activity, stability, and substrate selectivity. Simultaneous modification of substrate-binding and cation-binding sites can facilitate the release of hydrolysis products in double mutants, thereby improving degradation efficiency. Applications of artificial intelligence, like machine learning, support enzyme design and activity enhancement [156]. Synthetic biology extends optimization from isolated enzymes to engineered whole-cell chassis and synthetic microbial communities. Chassis engineering, pathway introduction, transporter mutation, adaptive evolution, and engineered expression of plastic hydrolases can be combined to metabolize polymer monomers and promote plastic degradation. Synthetic microbial communities can further improve plastic biodegradation through division of labor among engineered strains. In addition, carbon-based materials, nanomaterials, porous carriers, hydrophobins, polymer-binding modules, and functional peptides can serve as carriers or interface-regulation tools to enhance enzyme activity and stability of microbial systems, thereby improving biodegradation efficiency. In the future, biodegradation enhancement and material design should be optimized together.

### 8.3. Plastic Upcycling

The value of plastic biodegradation extends beyond simple substrate breakdown and should be further coupled with plastic recycling and resource valorization in future research. A comprehensive understanding of polymer chain scission, oligomer or monomer release, and microbial metabolic pathways could enable the conversion of degradation intermediates into value-added chemicals, bio-based materials, energy carriers, or agricultural feedstocks. Such conversion could be facilitated by integrating enzyme engineering, synthetic biology, and synthetic microbial consortia. For refractory plastics, combining pretreatment with microbial degradation could enhance polymer accessibility and microbial utilization. Additional efforts are required to advance product separation, carbon tracing, and life cycle analysis. Accordingly, future research should move beyond the sole pursuit of higher degradation efficiency and establish an integrated framework encompassing degradation, bioconversion, and safety assessment to achieve full valorization of waste plastics.

## 9. Conclusions

This review systematically summarizes recent progress in microbial plastic biodegradation, including polymer characteristics, major microbial groups, assessment methods, degradation mechanisms, and optimization strategies. It links differences in polymer structure with microbial degradation pathways and the selection of assessment methods. Microbial attachment and biofilm formation, enzymatic hydrolysis or oxidation, and subsequent metabolism and mineralization together provide a framework for understanding plastic biodegradation. Physicochemical characterization should be combined with biochemical validation and abiotic controls to distinguish microbial degradation from abiotic changes. Surface damage or weight loss alone cannot confirm mineralization. Pretreatment, bioaugmentation, and regulation of environmental parameters can improve biodegradation efficiency. However, polymer crystallinity, limitations in mass transfer, and variable environmental conditions remain barriers to practical application. The formation of micro- and nanoplastics, release of additives and intermediates, and biosafety of introduced microorganisms also require careful assessment. Future research should combine standardized evaluation, enzyme engineering and synthetic biology with plastic upcycling and ecological safety. Microbial biodegradation should therefore serve as a complementary approach to mechanical recycling and pyrolysis, with the choice depending on polymer properties and waste characteristics.

## Figures and Tables

**Table 1:** Representative microorganisms involved in plastic biodegradation.

Microorganisms	Genus	Representative Strain	Culture Condition	Degradation Efficiency	Plastic Type	Degradation Mechanism
Bacteria	*Alicycliphilus*	*Alicycliphilus denitrificans* BQ1 [32]	MSM; pH7; 37 °C; 48 h	85% Impranil clearing	PU	Biofilm formation facilitates bacterial attachment to the PU surface, after which extracellular hydrolytic enzymes cleave susceptible ester bonds in polyester-based PU. Urethane/carbamate linkages may also be disrupted, generating lower-molecular-weight oligomers and monomers that can be further assimilated and metabolized.
	*Stenotrophomonas*	*Stenotrophomonasindicatrix* [49]	LCFB; 28 d	N/A	PS	Biofilm formation facilitates bacterial attachment to the PS surface, followed by oxidative modification of the carbon backbone and/or aromatic rings. This oxidation can promote chain scission and generate lower-molecular-weight aromatic intermediates, which may subsequently be assimilated and metabolized through aromatic-compound degradation pathways.
		*Stenotrophomonas maltophilia* [50]	PS-containing mineral medium; 30 °C; 60 d	9.5%		
	*Phanerochaete*	*Phanerochaete chrysosporium* BKMF-1767 [51]	LC-PDA; 27 °C; 35 d	7%		
	*Paenibacillus*	*Paenibacillus lautus* [52]	LCFBM; 24 °C; 60 d	3.73%		
		*Paenibacillus* sp. [53]	BHM; pH7; 37 °C; 20 d	6%	PP	Biofilm formation facilitates bacterial attachment to the hydrophobic PP surface, followed by oxidative modification of the methyl-substituted C–C backbone. The introduction of carbonyl and hydroxyl groups can increase surface polarity and promote chain scission into lower-molecular-weight oxygenated fragments, which may subsequently be assimilated and metabolized.
	*Aeromonas*	*Aeromonas* sp. [53]		5%		
	*Acinetobacter*	*Acinetobacter baumannii* [54]	MSM; 30 °C; 50 d	33.3%		
Bacteria	*Acinetobacter*	*Acinetobacter baumannii* [54]	MSM; 30 °C; 50 d	27.1%	PVC	Biofilm formation facilitates bacterial colonization of the PVC surface, followed by oxidative modification of the chlorinated C–C backbone. Oxidation may promote chain scission and dechlorination, generating lower-molecular-weight products that can subsequently be assimilated and metabolized.
	*Neobacillus*	*Neobacillus* sp. PVCKKU3 [55]	MSM; pH7.5; 37 °C; 35 d	7.04%		
	*Ideonella*	*Ideonella sakaiensis* [34]	YSVM; 30 °C; 6 weeks	0.13 mg cm^−2^ day^−1^	PET	Bacterial degradation of PET is initiated by extracellular hydrolases that cleave ester bonds in the polyester backbone, leading to chain shortening and the release of soluble oligomers and intermediates such as BHET and MHET, and ultimately terephthalic acid and ethylene glycol. These low-molecular-weight products can then be transported into cells and further metabolized through central metabolic pathways.
	*Pseudomonas*	*Pseudomonas* sp. [16]	MSM; pH7; 30 °C; 28 d	N/A		
		*Pseudomonas putida* [43]	Nutrient broth; 37 °C; 30 d	22.86%		
		*Pseudomonas* sp. SH5B OP811462, *Pseudomonas aeruginosa* SH6B OP811485 [44]	MSM; 37 °C; 120 d	25%	PE	Biofilm formation promotes bacterial attachment to the hydrophobic PE/LDPE/HDPE surface, followed by oxidative modification of the non-hydrolysable C–C backbone. The introduction of carbonyl and hydroxyl groups can increase surface polarity and facilitate chain scission, generating lower-molecular-weight oxygenated fragments. These products may subsequently be assimilated and metabolized through fatty-acid-related pathways, including β-oxidation and central metabolism.
	*Achromobacter*	*Achromobacter xylosoxidans* [56]	Synthetic medium; pH7; 33.3 °C; 60 d	N/A		
	*Proteus*	*Proteus mirabilis* MN124173.1 [57]	MSM; 30 °C; 78 d	89.72%	LDPE	
		*Proteus mirabilis* KY027145.1 [57]		71.8%		
	*Marinobacter*	*Marinobacter* sp. [58]	Bushnell–Haas broth medium; 30 °C; 90 d	1.68%		
	*Rhodococcus*	*Rhodococcus pyridinivorans* strain UCC0018 [47]	Nutrient broth; 24 h	5.2%		
Bacteria	*Rhodococcus*	*Rhodococcus erythropolis* [48]	M9X; 25 °C; 80 d	N/A		
	*Paenarthrobacter*	*Paenarthrobacter nitroguajacolicus* [48]				
	*Brevibacillus*	*Brevibacillus parabrevis* CGK45 MW965577 [59]	MSM; 35 °C; 90 d	4%	HDPE	
	*Bacillus*	*Bacillus amyloliquefaciens* [60]	N/A	N/A	LDPE	
		*Bacillus subtilis* AP-04 OR288581 [46]	MSM; 37 °C; 60 d	36.55%		
		*Bacillus cereus* NJD1 [45]	CLMSM; pH7.2; 25 °C; 120 d	43%		
		*Bacillus cereus* SHBF2 [17]	MSM; pH7; 30 °C; 60 d	PP 6.77%; PE 6.87%	PP, PE	Biofilm formation promotes bacterial attachment to the hydrophobic surfaces of both PP and PE, followed by oxidative modification of their non-hydrolysable C–C backbones. For PE, oxidation introduces carbonyl and hydroxyl groups along the relatively unsubstituted carbon chain, facilitating chain scission and the formation of lower-molecular-weight oxygenated fragments. For PP, pendant methyl groups create tertiary carbon sites that are relatively more susceptible to oxidative attack, while crystallinity and structural complexity may restrict degradation. Oxidative modification subsequently facilitates chain scission and microbial assimilation.
Fungi	*Phanerochaete*	*Phanerochaete chrysosporium* BKMF-1767 [51]	LC-PDA; 27 °C; 35 d	7%	PS	Fungal hyphae colonize the PS/EPS surface and promote oxidative modification of the carbon backbone and/or aromatic rings through extracellular oxidative activity. This oxidation may facilitate chain scission and generate lower-molecular-weight aromatic intermediates that can subsequently be assimilated and metabolized.
	*Phellinus*	*Phellinus noxius* [61]	EPS added with 7 d old white rot fungi; Room temperature; 30 d	16%	EPS	
	*Coriolopsis*	*Coriolopsis gallica* MH855593.1 [15]	BHM; pH5.5; 28 °C; 60 d	19%	PVC	Fungal hyphae colonize the PVC surface and promote oxidative modification of the chlorinated C–C backbone through extracellular oxidative activity. This process may facilitate chain scission and possible dechlorination, generating lower-molecular-weight products that can subsequently be assimilated and metabolized.
	*Aspergillus*	*Aspergillus niger* KF638509.1 [15]		25.3%		
		*Aspergillus flavus* MW084630.1 [15]		23.6%		
		*Aspergillus glaucus* NG_063391.1 [62]	MSM; pH8.5; 37 °C; 28 d	32%		
				25%	PE	Fungal hyphae colonize the hydrophobic PE/LDPE/LLDPE surface and secrete extracellular oxidative enzymes that promote oxidation of the non-hydrolysable C–C backbone. The formation of carbonyl and hydroxyl groups can increase surface polarity and facilitate chain scission, generating lower-molecular-weight oxygenated fragments. These products may subsequently be assimilated and metabolized through fatty-acid-related pathways, including β-oxidation and central metabolism.
		*Aspergillus niger* NG_065763.1 [62]		40%		
	*Rhodotorula*	*Rhodotorula mucilaginosa* [63]	sterile seawater; 25 °C; 5 d	3.8%		
	*Schizophyllum*	*Schizophyllum commune* [64]	PDB; Room temperature; 30 d	1.18%	LLDPE	
	*Lentinus*	*Lentinus sajor-caju* TBRC6266 [64]		0.96%		
	*Neopestalotiopsis*	*Neopestalotiopsis phangngaensis* [19]	MSM; room temperature; 90 d	54.34%	LDPE	
	*Fusarium*	*Fusarium solani* MW216969.1 [65]	MSMA; 26 °C; 45 d	20.83%		
Fungi	*Penicillium*	*Penicillium corylophilum* CNMN-FP-04 [66]	Czapek’s agar with glucose; pH5.5; 28 °C; 30 d	6.06%		
		*Penicillium simplicissimum* [67]	MSM; 3 months	7.7%	PE	
		*Penicillium simplicissimum* [68]	*P. simplicissimum* culture medium; 30 °C; 28 d	3.09%	PET	Fungal PET degradation is initiated by extracellular hydrolases that cleave ester bonds, shortening polymer chains and releasing soluble oligomers and monomers. These products, including terephthalic acid and ethylene glycol, are assimilated and metabolized by fungal cells.
	*Fusarium*	*Fusarium oxysporum* [69]	N/A; 30 d	22%		
	*Cryptococcus*	*Cryptococcus flavus GB-1* [70]	emulsified PBSA agar containing soybean oil; 30 °C	N/A	PBSA	Fungal degradation of PBSA and PBAT is mainly initiated by extracellular hydrolases that cleave ester bonds in the polyester backbone, leading to chain scission and the release of lower-molecular-weight oligomers and monomers. These hydrolysis products may subsequently be assimilated and further metabolized by fungal cells.
	*Moesziomyces*	*Moesziomyces antarcticus* [71]	P-emulsified agar medium	N/A		
				N/A	PBAT	
Algae	*Uronema*	*Uronema africanum* [72]	BBM; 25 °C; 30 d	N/A	LDPE	Algal attachment and biofilm formation promote close contact with the hydrophobic LDPE surface. Extracellular metabolites and reactive oxygen species may induce oxidative modification of the non-hydrolysable C–C backbone, increasing carbonyl and hydroxyl groups and potentially facilitating chain scission into lower-molecular-weight oxygenated fragments.

## Data Availability

The original contributions presented in the study are included in the article/Supplementary Materials; further inquiries can be directed to the corresponding authors.

## Supplementary Materials

The following supporting information can be downloaded at: https://www.mdpi.com/article/10.3390/microorganisms14102335/s1, Figure S1: The flow diagram for literature filtering; Table S1: The inclusion and exclusion criteria for publications; Text S1: Literature searching and filtering description.

## Abbreviations

The following abbreviations are used in this manuscript:

PE	Polyethylene
PET	Polyethylene terephthalate
PP	Polypropylene
PVC	Polyvinyl chloride
SEM	Scanning electron microscopy
FTIR	Fourier transform infrared spectroscopy
PS	Polystyrene
PLA	Polylactic acid
PU	Polyurethane
PEG	Polyethylene glycol
LDPE	Low-density polyethylene
HDPE	High-density polyethylene
LLDPE	Linear low-density polyethylene
MHET	Mono(2-hydroxyethyl) terephthalate
BHET	Bis(2-hydroxyethyl) terephthalate
EPS	Expanded polystyrene
LCFBM	Lignin-Cellulose Fermentation Basal Medium
MSM	Mineral Salt Medium
M9X	M9 minimal salts medium
BHM	Bushnell-Haas Medium
YSV	Yeast extract–sodium carbonate–vitamin medium
CLMSM	Cellulose-Lignin Mineral Salt Medium
MSMA	Mineral Salt Medium Agar
PDB	Potato Dextrose Broth
LC-PDA	Lignocellulose Potato Dextrose Agar
BBM	Bold’s Basal Medium
QCM-D	Quartz crystal microbalance dissipative technology
TPA	Terephthalic acid
EG	Ethylene glycol
AlkB	Alkane hydroxylase
PCA	Protocatechuic acid

## References

[1] Gondwe G.G., Mtonga K., Mapoma H.W.T. (2026). Plastic waste dynamics and governance challenges in urban Malawi. Discov. Environ..

[2] Andrady A.L., Barnes P.W., Bornman J.F., Gouin T., Madronich S., White C.C., Zepp R.G., Jansen M.A.K. (2022). Oxidation and fragmentation of plastics in a changing environment; from UV-radiation to biological degradation. Sci. Total Environ..

[3] Darabi M., Majeed H., Diehl A., Norton J., Zhang Y. (2021). A Review of Microplastics in Aquatic Sediments: Occurrence, Fate, Transport, and Ecological Impact. Curr. Pollut. Rep..

[4] Nguyen M.-K., Rakib M.R.J., Hwangbo M., Kim J. (2025). Microplastic accumulation in soils: Unlocking the mechanism and biodegradation pathway. J. Hazard. Mater. Adv..

[5] Chen H., Huang D., Zhou W., Deng R., Yin L., Xiao R., Li S., Li F., Lei Y. (2024). Hotspots lurking underwater: Insights into the contamination characteristics, environmental fates and impacts on biogeochemical cycling of microplastics in freshwater sediments. J. Hazard. Mater..

[6] Schyns Z.O.G., Shaver M.P. (2021). Mechanical Recycling of Packaging Plastics: A Review. Macromol. Rapid Commun..

[7] Schade A., Melzer M., Zimmermann S., Schwarz T., Stoewe K., Kuhn H. (2024). Plastic Waste Recycling—A Chemical Recycling Perspective. ACS Sustain. Chem. Eng..

[8] Luo H., Tyrrell H., Bai J., Ibrahim Muazu R., Long X. (2024). Fundamental, technical and environmental overviews of plastic chemical recycling. Green Chem..

[9] Zhang M., Qi Y., Zhang W., Wang M., Li J., Lu Y., Zhang S., He J., Cao H., Tao X. (2024). A review on waste tires pyrolysis for energy and material recovery from the optimization perspective. Renew. Sustain. Energy Rev..

[10] Bassyouni M., Nasser R., El-Bagoury M., Shaker I., Attia A.M., Elhenawy Y., Aboelela D. (2025). Integrating medical plastic waste pyrolysis and circular economy for environmental sustainability. Renew. Sustain. Energy Rev..

[11] Choi J., Kim H., Ahn Y.-R., Kim M., Yu S., Kim N., Lim S.Y., Park J.-A., Ha S.-J., Lim K.S. (2024). Recent advances in microbial and enzymatic engineering for the biodegradation of micro- and nanoplastics. RSC Adv..

[12] Harrat R., Bourzama G., Burgaud G., Coton E., Bourezgui A., Soumati B. (2024). Assessing the Biodegradation of Low-Density Polyethylene Films by *Candida tropicalis* SLNEA04 and *Rhodotorula mucilaginosa* SLNEA05. Diversity.

[13] Sun X., Chen Z., Kong T., Chen Z., Dong Y., Kolton M., Cao Z., Zhang X., Zhang H., Liu G. (2022). Mycobacteriaceae Mineralizes Micropolyethylene in Riverine Ecosystems. Environ. Sci. Technol..

[14] da Silva M.R.F., Souza K.S., Motteran F., de Araújo L.C.A., Singh R., Bhadouria R., de Oliveira M.B.M. (2024). Exploring biodegradative efficiency: A systematic review on the main microplastic-degrading bacteria. Front. Microbiol..

[15] Alhajar M.W., Al-Ahmady K.K., Sabri Mhemid R.K., Mhemid R. (2024). Fungal Biodegradation of Polyvinyl Chloride in Aqueous Environments. Egypt. J. Aquat. Biol. Fish..

[16] Aryal S., Shrestha S., Acharya A., Paudel S.R. (2025). Screening of selected microbial isolates for plastic degradation: A batch study. Int. J. Environ. Stud..

[17] Hossain S., Shukri Z.N.A., Waiho K., Ibrahim Y.S., Kamaruzzan A.S., Rahim A.I.A., Draman A.S., Wahab W., Khatoon H., Kasan N.A. (2024). Biodegradation of polyethylene (PE), polypropylene (PP), and polystyrene (PS) microplastics by floc-forming bacteria, *Bacillus cereus* strain SHBF2, isolated from a commercial aquafarm. Environ. Sci. Pollut. Res..

[18] Wang D., Yang Y., Ye Y., He D., Fu Q., Liu X. (2026). Microbial Degradation of Plastics: Mechanisms, Pathways, and Multiomics Insights. Environ. Sci. Technol..

[19] Khruengsai S., Sripahco T., Pripdeevech P. (2022). Microbial degradation of low-density polyethylene by *Neopestalotiopsis phangngaensis*. J. Gen. Appl. Microbiol..

[20] White E.M., Horn J., Wang S., Crawford B., Ritchie B.W., Carraway D., Locklin J. (2021). Comparative Study of the Biological Degradation of Poly(3-Hydroxybutyrate-co-3-Hydroxyhexanoate) Microbeads in Municipal Wastewater in Environmental and Controlled Laboratory Conditions. Environ. Sci. Technol..

[21] Solanki A., Sharma V., Sharma P., Kumar D. (2026). Current trends in microplastic removal using biodegradation approaches and advancement—A review. J. Ind. Eng. Chem..

[22] Lomwongsopon P., Martínez B.M., Jiménez A.B., Bardenstein A.L., Kusano Y., de Claville Christiansen J., Varrone C. (2025). Enhancing biodegradation of polyolefins and real mixed plastic waste by combination of pretreatment and mixed microbial consortia. Chemosphere.

[23] Hwang D.-H., Lee M.-E., Kim M., Jeong W.-Y., Hyeon J.E., Shi X., Han S.O. (2025). Surface-immobilized microalgal biohybrid system with dual-specificity enzyme scaffold for enhanced PET degradation and adaptive algal responses in aquatic environments. J. Water Process Eng..

[24] Tournier V., Topham C.M., Gilles A., David B., Folgoas C., Moya-Leclair E., Kamionka E., Desrousseaux M.-L., Texier H., Gavalda S. (2020). An engineered PET depolymerase to break down and recycle plastic bottles. Nature.

[25] Kučić Grgić D., Cvetnić M., Bolanča T., Markić M., Ukić Š., Bule K., Prevarić V., Ocelić Bulatović V., Kovačević A., Lovrinčić E. (2021). Bioremediation of MP-polluted Waters Using Bacteria *Bacillus licheniformis*, *Lysinibacillus massiliensis*, and Mixed Culture of *Bacillus* sp. and *Delftia acidovorans*. Chem. Biochem. Eng. Q..

[26] Romano I., Ventorino V., Schettino M., Magaraci G., Pepe O. (2024). Changes in Soil Microbial Communities Induced by Biodegradable and Polyethylene Mulch Residues Under Three Different Temperatures. Microb. Ecol..

[27] Mohanan N., Montazer Z., Sharma P.K., Levin D.B. (2020). Microbial and Enzymatic Degradation of Synthetic Plastics. Front. Microbiol..

[28] Danso D., Chow J., Streit W.R. (2019). Plastics: Environmental and Biotechnological Perspectives on Microbial Degradation. Appl. Environ. Microbiol..

[29] Santo M., Weitsman R., Sivan A. (2013). The role of the copper-binding enzyme—Laccase—In the biodegradation of polyethylene by the actinomycete *Rhodococcus ruber*. Int. Biodeterior. Biodegrad..

[30] Kim H.-W., Jo J.H., Kim Y.-B., Le T.-K., Cho C.-W., Yun C.-H., Chi W.S., Yeom S.-J. (2021). Biodegradation of polystyrene by bacteria from the soil in common environments. J. Hazard. Mater..

[31] Khandare S.D., Chaudhary D.R., Jha B. (2025). Isolation and purification of esterase enzyme from marine bacteria associated with biodegradation of polyvinyl chloride (PVC). Biodegradation.

[32] Fuentes-Jaime J., Vargas-Suárez M., Cruz-Gómez M.J., Loza-Tavera H. (2022). Concerted action of extracellular and cytoplasmic esterase and urethane-cleaving activities during Impranil biodegradation by *Alicycliphilus denitrificans* BQ1. Biodegradation.

[33] Kossentini Kallel T., Houichi H. (2015). Assessment of Biodegradation of PLA/PCL and PLA/PEG Biopolymers under Aerobic and Anaerobic Conditions. J. Mater. Environ. Sci..

[34] Yoshida S., Hiraga K., Takehana T., Taniguchi I., Yamaji H., Maeda Y., Toyohara K., Miyamoto K., Kimura Y., Oda K. (2016). A bacterium that degrades and assimilates poly(ethylene terephthalate). Science.

[35] Han X., Liu W., Huang J.-W., Ma J., Zheng Y., Ko T.-P., Xu L., Cheng Y.-S., Chen C.-C., Guo R.-T. (2017). Structural insight into catalytic mechanism of PET hydrolase. Nat. Commun..

[36] Wei R., von Haugwitz G., Pfaff L., Mican J., Badenhorst C.P.S., Liu W., Weber G., Austin H.P., Bednar D., Damborsky J. (2022). Mechanism-Based Design of Efficient PET Hydrolases. ACS Catal..

[37] Hosoya H., Miyazaki N., Sugisaki Y., Takanashi E., Tsurufuji M., Yamasaki M., Tamura G. (1978). Bacterial Degradation of Synthetic Polymers and Oligomers with the Special Reference to the Case of Polyethylene Glycol. Agric. Biol. Chem..

[38] Thomsen T.B., Almdal K., Meyer A.S. (2023). Significance of poly(ethylene terephthalate) (PET) substrate crystallinity on enzymatic degradation. New Biotechnol..

[39] Wei R., Westh P., Weber G., Blank L.M., Bornscheuer U.T. (2025). Standardization guidelines and future trends for PET hydrolase research. Nat. Commun..

[40] Thomsen T.B., Hunt C.J., Meyer A.S. (2022). Influence of substrate crystallinity and glass transition temperature on enzymatic degradation of polyethylene terephthalate (PET). New Biotechnol..

[41] Gorish B.M.T., Abdelmula W.I.Y., Sethupathy S., Dar M.A., Shahnawaz M., Zhu D. (2024). Microbial degradation of polyethylene polymer: Current paradigms, challenges, and future innovations. World J. Microbiol. Biotechnol..

[42] Fortaleza J.A.G., Asemoloye M.D., Tekere M., Chigwada A.D., Zhou X., Sabeeh M., Cabuhat K.S.P., De Jesus R.S. (2026). Microbial and enzymatic biodegradation of synthetic plastics: Mechanisms, environmental constraints, and translational limits. Waste Manag. Bull..

[43] Hassan I.A. (2022). Biodegradation of Polyethylene Terephthalate (PET Bottle) by Bacteria Isolated from Elepe Dumpsite at Ikorodu, Lagos State, Nigeria. J. Res. Rev. Sci..

[44] Ali S., Rehman A., Hussain S.Z., Bukhari D.A. (2023). Characterization of plastic degrading bacteria isolated from sewage wastewater. Saudi J. Biol. Sci..

[45] Jayan N., Skariyachan S., Sebastian D. (2023). The escalated potential of the novel isolate *Bacillus cereus* NJD1 for effective biodegradation of LDPE films without pre-treatment. J. Hazard. Mater..

[46] Akash K., Parthasarathi R., Elango R., Bragadeeswaran S. (2025). Exploring the plastic-fed Indian mealworm (*Tenebrio molitor*) gut bacterial strain (*Bacillus subtilis* AP-04)—A potential driver of polyethylene degradation. J. Hazard. Mater..

[47] Abdullah H., Ahmad M.F., Maniyam M.N., Azman H.H., Yaacob N.S. (2023). Potential of Rhodococcus Pyridinovoran Strain UCC0018 as the Plastic Waste Degradation Agent for Green Technology.

[48] Cabigliera S.B., Gaudioso D., Chelazzi D., Conti L., Muzzi B., Cantagalli D., Cincinelli A., Tegli S., Jenkins A., Martellini T. (2025). Isolation of a novel microplastic-degrading bacterial strain: A promising agent for low-density polyethylene remediation. Chemosphere.

[49] Zarra F., Funari R., Cucini C., Nardi F., Carapelli A., Marri L., Frati F. (2025). Novel insights into insect mediated polystyrene biodegradation through bacterial genome analyses. Sci. Rep..

[50] Xiang P., Zhang Y., Zhang T., Wu Q., Zhao C., Li Q. (2023). A novel bacterial combination for efficient degradation of polystyrene microplastics. J. Hazard. Mater..

[51] Guo Z., Zha Y., Guo X., Ling X., Yao L., Han L., Yang F. (2026). Mechanistic Insights into UV/H_2_O_2_-Aged Polystyrene Enable an Optimized Protocol for Fungal Biodegradation. J. Environ. Chem. Eng..

[52] de Souza E.P., Sebestyen-França H., Basaglia M.V., Watson A., Ferro M., Bettini S.H.P., Henrique-Silva F. (2026). *Paenibacillus lautus* isolated from the *Sphenophorus levis* gut causes structural and physicochemical changes on polystyrene surface. Front. Microbiol..

[53] Wahyuningsih C., Zainuri M., Ismanto A., Indarjo A., Kusumaningrum H.P., Hadibarata T., Herida A.P., Safinatunnajah N. (2026). Biodegradation of polypropylene by *Paenibacillus* sp. and *Aeromonas* sp. isolated from the Mojo Mangrove Forest Essential Ecosystem Area, Indonesia. Mar. Pollut. Bull..

[54] Bansal M., Santhiya D., Sharma J.G. (2025). Simulated dump yard microbes drive significant biodegradation of polypropylene and polyvinyl chloride microplastics. J. Hazard. Mater..

[55] Choonut A., Wongfaed N., Poolpol A., Jumpa T., Boonlue S., Wantala K., Pinyakong O., Reungsang A., Plangklang P., Sittijunda S. (2026). Unveiling a novel *Neobacillus* strain: Optimization, metabolomic, and genomic insights into polyvinyl chloride microplastic biodegradation. J. Environ. Chem. Eng..

[56] Tiwari N., Santhiya D., Sharma J.G. (2024). Significance of landfill microbial communities in biodegradation of polyethylene and nylon 6,6 microplastics. J. Hazard. Mater..

[57] Akhigbe G.E., EnochOghene A.E., Olumurewa K.O., Koleoso O.B., Ogbonna N.D. (2024). Characterization of low-density polyethylene (LDPE) films degraded using bacteria strains isolated from oil-contaminated soil. Environ. Technol..

[58] Khandare S.D., Agrawal D., Mehru N., Chaudhary D.R. (2022). Marine bacterial based enzymatic degradation of low-density polyethylene (LDPE) plastic. J. Environ. Chem. Eng..

[59] Gupta K.K., Chandra H., Sagar K., Sharma K.K., Devi D. (2023). Degradation of high density polyethylene (HDPE) through bacterial strain from Cow faeces. Biocatal. Agric. Biotechnol..

[60] Das M.P., Kumar S. (2013). Influence of Cell Surface Hydrophobicity in Colonization and Biofilm Formation on LDPE Biodegradation. Int. J. Pharm. Pharm. Sci..

[61] Karimah S.N., Yanto D.H.Y., Rahayu G., Nurhayat O.D. (2025). The potential of White Rot fungi from Indonesia for biodegradation of expanded polystyrene. Bioremediat. J..

[62] Saeed S., Iqbal A., Deeba F. (2022). Biodegradation study of Polyethylene and PVC using naturally occurring plastic degrading microbes. Arch. Microbiol..

[63] Vaksmaa A., Polerecky L., Dombrowski N., Kienhuis M.V.M., Posthuma I., Gerritse J., Boekhout T., Niemann H. (2023). Polyethylene degradation and assimilation by the marine yeast *Rhodotorula mucilaginosa*. ISME Commun..

[64] Aiduang W., Jatuwong K., Ratanapong K., Promjaidee T., Xayyavong O., Hongsanan S., Lumyong S. (2025). Breaking Down Linear Low-Density Polyethylene (LLDPE) Using Fungal Mycelium (Part A): A Path Towards Sustainable Waste Management and Its Possible Economic Impacts. Life.

[65] Munir E., Pasaribu Y., Mubtasima S., Nasution A.F. (2024). The Diversity of Fungi in Landfill and their Potential to Degrade Plastic. OnLine J. Biol. Sci..

[66] Rastimesina I., Postolachi O., Josan V., Cotoman A., Mamaliga V. (2021). Screening of Low Density Polyethylene Degrading Microorganisms.

[67] Sowmya H.V., Ramalingappa, Krishnappa M., Thippeswamy B. (2015). Degradation of polyethylene by *Penicillium simplicissimum* isolated from local dumpsite of Shivamogga district. Environ. Dev. Sustain..

[68] Moyses D.N., Teixeira D.A., Waldow V.A., Freire D.M.G., Castro A.M. (2021). Fungal and enzymatic bio-depolymerization of waste post-consumer poly(ethylene terephthalate) (PET) bottles using *Penicillium* species. 3 Biotech.

[69] Tertyshnaya Y.V., Pozdnyakova N.N., Podzorova M.V. (2025). Impact of soil micromycetes on the structural characteristics of polyethylene terephthalate. Russ. Chem. Bull..

[70] Watanabe T., Suzuki K., Shinozaki Y., Yarimizu T., Yoshida S., Sameshima-Yamashita Y., Koitabashi M., Kitamoto H.K. (2015). A UV-induced mutant of *Cryptococcus flavus* GB-1 with increased production of a biodegradable plastic-degrading enzyme. Process Biochem..

[71] Tanaka E., Koitabashi M., Kitamoto H. (2019). A teleomorph of the ustilaginalean yeast *Moesziomyces antarcticus* on barnyardgrass in Japan provides bioresources that degrade biodegradable plastics. Antonie Van. Leeuwenhoek.

[72] Sanniyasi E., Gopal R.K., Gunasekar D.K., Raj P.P. (2021). Biodegradation of low-density polyethylene (LDPE) sheet by microalga, *Uronema africanum* Borge. Sci. Rep..

[73] Kosiorowska K.E., Połomska X., Wang G., Borodina I., Mirończuk A.M. (2021). Efficient biodegradation of aliphatic polyester by genetically engineered strains of the yeast *Yarrowia lipolytica*. Int. Biodeterior. Biodegrad..

[74] Buron-Moles G., Vandenbossche V., Gorret N., Santonja-Blasco L., González-Aranda T., Cameleyre X., Guillouet S. (2025). Biodegradation of pre-treated low-density polyethylene (LDPE) by *Yarrowia lipolytica* determined by oxidation and molecular weight reduction. Polym. Degrad. Stab..

[75] Alami N.H., Puteri Z.R., Esaki T., Sugai Y., Pratama F., Purwasena I.A., Aditiawati P. (2025). Functional and genomic characterization of polyethylene degrading yeast *Meyerozyma carpophila* M6.0.2 isolated from marine plastic debris in East Java Indonesia. Sci. Rep..

[76] Rajan A., Ameen F., Jambulingam R., Shankar V. (2024). Biodegradation of Polyurethane by Fungi Isolated from Industrial Wastewater—A Sustainable Approach to Plastic Waste Management. Polymers.

[77] Fan X., Kong L., Wang J., Tan Y., Xu X., Li M., Zhu L. (2024). Surface-programmed microbiome assembly in phycosphere to microplastics contamination. Water Res..

[78] Han Y.-N., Wei M., Han F., Fang C., Wang D., Zhong Y.-J., Guo C.-L., Shi X.-Y., Xie Z.-K., Li F.-M. (2020). Greater Biofilm Formation and Increased Biodegradation of Polyethylene Film by a Microbial Consortium of *Arthrobacter* sp. and *Streptomyces* sp.. Microorganisms.

[79] Liu W., Lu L., Zeng X., Zhang T., Ma L. (2026). Microbial synergism in a synthetic consortium enabled efficient polyethylene biodegradation. Ecotoxicol. Environ. Saf..

[80] Jiao H., Xiong M., Al-Tohamy R., Schagerl M., Kornaros M., Ali S.S. (2026). Rethinking plastics through microbial biodegradation and circular economy innovation—A review. Environ. Chem. Ecotoxicol..

[81] El-Naggar M.M., El-sheekh M., El-Shanshoury A.E.R.R., Yousry F.M. (2024). Biodegradation of Low-Density Polyethylene Plastic Using Marine Bacterial Consortium. Egypt. J. Aquat. Biol. Fish..

[82] Zhang Y., Ma J., O’Connor P., Zhu Y.-G. (2022). Microbial communities on biodegradable plastics under different fertilization practices in farmland soil microcosms. Sci. Total Environ..

[83] Ahmed M., Iram S., Tabassum N., Sajid M., Paseutsakoun K., Aleksza L., Székács A. (2025). Biodegradation Efficacy of *Aspergillus niger* and *Trichoderma harzianum* on Low-Density Polyethylene. Polymers.

[84] Sutkar P.R., Hadkar S.S., Kamble S.A., Dhulap V.P. (2025). Biodegradation potential of low-density polyethylene (LDPE) using *Aspergillus niger* and *Phanerochaete chrysosporium*. Discov. Environ..

[85] LeviRam I., Gross A., McCarthy D., Herzberg M. (2019). Real-time analysis of atrazine biodegradation and sessile bacterial growth: A quartz crystal microbalance with dissipation monitoring study. Chemosphere.

[86] Zhang Z., Fan X., Zhang R., Pan X., Zhang X., Ding Y., Liu Y. (2025). Biodegradation characterization and mechanism of low-density polyethylene by the enriched mixed-culture from plastic-contaminated soil. J. Hazard. Mater..

[87] Taipale S.J., Vesamäki J., Kautonen P., Kukkonen J.V.K., Biasi C., Nissinen R., Tiirola M. (2023). Biodegradation of microplastic in freshwaters: A long-lasting process affected by the lake microbiome. Environ. Microbiol..

[88] Reviakine I., Johannsmann D., Richter R.P. (2011). Hearing What You Cannot See and Visualizing What You Hear: Interpreting Quartz Crystal Microbalance Data from Solvated Interfaces. Anal. Chem..

[89] Tao H., Yang L., Qi Y., Chen Y., Yu D., Zhou L., Lin T., Xu H., Song J. (2022). Deposition of polystyrene microplastics on bare or biofilm-coated silica analysed via QCM-D. Sci. Total Environ..

[90] Obrador-Viel T., Zadjelovic V., Nogales B., Bosch R., Christie-Oleza J.A. (2024). Assessing microbial plastic degradation requires robust methods. Microb. Biotechnol..

[91] Robertson D., Kaundinya I. Influence of Transition Metal Ions on the Thermo-Oxidative Degradation of Polyethylene Geomembranes (GBR-P) Used in Road Tunnels. Proceedings of the 10th International Conference on Geosynthetics.

[92] Tian L., Kolvenbach B., Corvini N., Wang S., Tavanaie N., Wang L., Ma Y., Scheu S., Corvini P.F.-X., Ji R. (2017). Mineralisation of 14C-labelled polystyrene plastics by *Penicillium variabile* after ozonation pre-treatment. New Biotechnol..

[93] Fernando S.S., Christensen P.A., Egerton T.A., White J.R. (2007). Carbon dioxide evolution and carbonyl group development during photodegradation of polyethylene and polypropylene. Polym. Degrad. Stab..

[94] Khatua S., Simal-Gandara J., Acharya K. (2024). Myco-remediation of plastic pollution: Current knowledge and future prospects. Biodegradation.

[95] Tsochatzis E., Lopes J., Gika H., Theodoridis G. (2020). Polystyrene Biodegradation by *Tenebrio molitor* Larvae: Identification of Generated Substances Using a GC-MS Untargeted Screening Method. Polymers.

[96] Zhang H., Yin C.-F., Song X., Zhou N.-Y., Xu Y. (2026). Polyvinyl chloride depolymerization and dechlorination by *Tenebrio molitor* larva-derived bacteria. J. Hazard. Mater..

[97] Erni-Cassola G., Wright R.J., Gibson M.I., Christie-Oleza J.A. (2020). Early Colonization of Weathered Polyethylene by Distinct Bacteria in Marine Coastal Seawater. Microb. Ecol..

[98] Dey S., Rout A.K., Behera B.K., Ghosh K. (2022). Plastisphere community assemblage of aquatic environment: Plastic-microbe interaction, role in degradation and characterization technologies. Environ. Microbiome.

[99] Gilan I., Sivan A. (2013). Effect of proteases on biofilm formation of the plastic-degrading actinomycete *Rhodococcus ruberC208*. FEMS Microbiol. Lett..

[100] Papenfort K., Bassler B.L. (2016). Quorum sensing signal–response systems in Gram-negative bacteria. Nat. Rev. Microbiol..

[101] Wang J., Lu L., Sun Y., Messer L.F., Wu M., Duan Z., Shi J., Yang Y., Li C., Mao Y. (2026). AHL-mediated quorum sensing drives plastisphere formation and elevates pathogenic potential. ISME J..

[102] Kumari S., Amaresan N. (2026). Bacterial enzymatic depolymerization of microplastics: Mechanisms, biofilm-mediated processes and environmental regulation. J. Environ. Chem. Eng..

[103] Chauhan D., Agrawal G., Deshmukh S., Roy S.S., Priyadarshini R. (2018). Biofilm formation by *Exiguobacterium* sp. DR11 and DR14 alter polystyrene surface properties and initiate biodegradation. RSC Adv..

[104] Zhang X., Zhou S., Jiang C., Wang X., Ma S., Zhuang X. (2026). Quorum sensing on microplastics shapes biofilm function in wastewater systems. Commun. Earth Environ..

[105] Dahmer B.R., de Lima J.C., Ruggiero Bachega J.F., Wymore T., Saraiva Macedo Timmers L.F. (2025). It Is Dangerous to Go Alone: Strategies to Optimize PET Biocatalysis and Upcycling through Enzymatic Synergism. ACS Omega.

[106] Palm G.J., Reisky L., Böttcher D., Müller H., Michels E.A.P., Walczak M.C., Berndt L., Weiss M.S., Bornscheuer U.T., Weber G. (2019). Structure of the plastic-degrading *Ideonella sakaiensis* MHETase bound to a substrate. Nat. Commun..

[107] Jones S.M., Solomon E.I. (2015). Electron transfer and reaction mechanism of laccases. Cell. Mol. Life Sci..

[108] Martínez A.T. (2002). Molecular biology and structure-function of lignin-degrading heme peroxidases. Enzym. Microb. Technol..

[109] Ong A., Teo J.Y.Q., Lim J.Y.C. (2024). Interfacial Reactions in Chemical Recycling and Upcycling of Plastics. ACS Appl. Mater. Interfaces.

[110] Zhou J., Cui Z., Wei R., Dong W., Jiang M. (2025). Interfacial catalysis in enzymatic PET plastic depolymerization. Trends Chem..

[111] Majumder D., Dutta A., Lahiri D., Nag M. (2026). Programmable biodegradation: Lipase-driven microplastic degradation via AI and omics. Prep. Biochem. Biotechnol..

[112] Wu H., Li H., Li Z., Liu X., Li Q., Cheng M., Gong J. (2025). Interfacial engineering-based colonization of biofilms on polyethylene terephthalate (PET) surfaces: Implications for whole-cell biodegradation of microplastics. Sci. Total Environ..

[113] Dos Santos L.F.O., Kagueyama S.S., Hilário I.d.B., Trindade A.R.d.F., Dos Santos Filho J.R., Peralta R.A., Moreira R.d.F.P.M., de Souza C.G.M., Simão R.d.C.G., Bracht A. (2026). An Evidence-Level Framework for Evaluating Enzyme-Mediated Plastic and Microplastic Transformation. Microorganisms.

[114] Zurier H.S., Goddard J.M. (2022). Directed Immobilization of PETase on Mesoporous Silica Enables Sustained Depolymerase Activity in Synthetic Wastewater Conditions. ACS Appl. Bio Mater..

[115] Roberts C., Edwards S., Vague M., León-Zayas R., Scheffer H., Chan G., Swartz N.A., Mellies J.L. (2020). Environmental Consortium Containing *Pseudomonas* and *Bacillus* Species Synergistically Degrades Polyethylene Terephthalate Plastic. mSphere.

[116] Pérez-Pantoja D., De la Iglesia R., Pieper D.H., González B. (2008). Metabolic reconstruction of aromatic compounds degradation from the genome of the amazing pollutant-degrading bacterium *Cupriavidus necator* JMP134. FEMS Microbiol. Rev..

[117] Pandey J.K., Kumar A.P., Singh R.P. (2003). Biodegradation of packaging materials: Composting of polyolefins. Macromol. Symp..

[118] Vaksmaa A., Vielfaure H., Polerecky L., Kienhuis M.V.M., van der Meer M.T.J., Pflüger T., Egger M., Niemann H. (2024). Biodegradation of polyethylene by the marine fungus *Parengyodontium album*. Sci. Total Environ..

[119] Lee B., Pometto A.L., Fratzke A., Bailey T.B. (1991). Biodegradation of Degradable Plastic Polyethylene by *Phanerochaete* and *Streptomyces* Species. Appl. Environ. Microbiol..

[120] Sheik S., Chandrashekar K.R., Swaroop K., Somashekarappa H.M. (2015). Biodegradation of gamma irradiated low density polyethylene and polypropylene by endophytic fungi. Int. Biodeterior. Biodegrad..

[121] Ji S.H., Yoo S., Park S., Lee M.J. (2024). Biodegradation of low-density polyethylene by plasma-activated *Bacillus* strain. Chemosphere.

[122] Hosseini F.S., Asoodeh A., Ostad Movahed S., Makhdoumi A. (2024). An integrated approach for plastic polymer degradation by the gut bacterial resident of superworm, *Zophobas morio* (Coleoptera:Tenebrionidae). Environ. Sci. Pollut. Res..

[123] Pacwa-Płociniczak M., Płaza G.A., Piotrowska-Seget Z., Cameotra S.S. (2011). Environmental Applications of Biosurfactants: Recent Advances. Int. J. Mol. Sci..

[124] Ma X., Han J., Ni P., Wang L., Zhou Y., Wu J., Wang Y. (2026). Aqueous micellar two-phase emulsions: An integrated reaction-separation platform for sustainable enzymatic depolymerization of PET waste. J. Hazard. Mater..

[125] Vimala P.P., Mathew L. (2016). Biodegradation of Polyethylene Using *Bacillus subtilis*. Procedia Technol..

[126] Taghavi N., Zhuang W.-Q., Baroutian S. (2022). Effect of rhamnolipid biosurfactant on biodegradation of untreated and UV-pretreated non-degradable thermoplastics: Part 2. J. Environ. Chem. Eng..

[127] Feng J., Wang Y., Lu Y., Artur C., Fu J. (2026). Mechanisms and optimization Strategies for surfactant-mediated enhancement of enzymatic hydrolysis of Poly(ethylene terephthalate). Environ. Res..

[128] Thakur V., Baghmare P., Verma A., Verma J.S., Geed S.R. (2024). Recent progress in microbial biosurfactants production strategies: Applications, technological bottlenecks, and future outlook. Bioresour. Technol..

[129] Kumar Sen S., Raut S. (2015). Microbial degradation of low density polyethylene (LDPE): A review. J. Environ. Chem. Eng..

[130] Jeyakumar D., Chirsteen J., Doble M. (2013). Synergistic effects of pretreatment and blending on fungi mediated biodegradation of polypropylenes. Bioresour. Technol..

[131] Khan M.S., Dhavan P.P., Ratna D., Sonawane S.S., Shimpi N.G. (2021). LDPE:PLA and LDPE:PLA:OMMT polymer composites: Preparation, characterization, and its biodegradation using *Bacillus* species isolated from dumping yard. Polym. Adv. Technol..

[132] Galgali P. (2004). Fungal degradation of carbohydrate-linked polystyrenes. Carbohydr. Polym..

[133] Soni R., Kapri A., Zaidi M.G.H., Goel R. (2009). Comparative Biodegradation Studies of Non-poronized and Poronized LDPE Using Indigenous Microbial Consortium. J. Polym. Environ..

[134] Patil R., Bagde U.S. (2016). Development of Novel Bacterial Strains for Enhanced Biodegradation of Plastic Polymers by Protoplast Fusion. Asian J. Microbiol. Biotechnol. Environ. Sci..

[135] Xiong Z., Chen X., Zou Z., Peng L., Zou L., Liu B., Li Q. (2025). Improving efficiency of bacterial degradation of polyethylene microplastics using atmospheric and room temperature plasma mutagenesis. Bioresour. Technol..

[136] Yan F., Wei R., Cui Q., Bornscheuer U.T., Liu Y. (2021). Thermophilic whole-cell degradation of polyethylene terephthalate using engineered *Clostridium thermocellum*. Microb. Biotechnol..

[137] Khampratueng P., Anal A.K. (2026). Enhancing the biodegradation of low-density polyethylene (LDPE) using novel bacterial consortia: *Bacillus* sp. AS3 and *Sphingobacterium* sp. AS8. J. Environ. Sci..

[138] Lee A., Mohd Sahari S.S.B., Liew M.S. (2021). Feasibility study of a co-culture system for PET-degrading bacteria to increase biodegradation performance. Bioremediat. J..

[139] Howard S.A., McCarthy R.R. (2023). Modulating biofilm can potentiate activity of novel plastic-degrading enzymes. npj Biofilms Microbiomes.

[140] Tribedi P., Das Gupta A., Sil A.K. (2015). Adaptation of *Pseudomonas* sp. AKS2 in biofilm on low-density polyethylene surface: An effective strategy for efficient survival and polymer degradation. Bioresour. Bioprocess..

[141] Pathak S., Ray A.K., Verma R.K., Kumar V. (2015). Optimization of the Temperature Dependent Biodegradation of Polythene. Asian J. Microbiol. Biotechnol. Environ. Sci..

[142] El-Dash H.A., Yousef N.E., Aboelazm A.A., Awan Z.A., Yahya G., El-Ganiny A.M. (2023). Optimizing Eco-Friendly Degradation of Polyvinyl Chloride (PVC) Plastic Using Environmental Strains of *Malassezia* Species and *Aspergillus fumigatus*. Int. J. Mol. Sci..

[143] Chen Z., Zhao W., Xing R., Xie S., Yang X., Cui P., Lü J., Liao H., Yu Z., Wang S. (2020). Enhanced in situ biodegradation of microplastics in sewage sludge using hyperthermophilic composting technology. J. Hazard. Mater..

[144] Masud Hossain S. (2006). Aerobic Biodepolymerization Studies of Polyethylene Using *Phanerochaete chrysosporium*. Indian J. Environ. Prot..

[145] Méndez C.R., Vergaray G., Béjar V.R., Cárdenas K.J. (2007). Isolation and Characterization of Polyethylene-Biodegrading Mycromycetes. Rev. Peru. Biol..

[146] Fang X., Cai Z., Wang X., Li M., Gong H., Yan M. (2024). Isolation and Identification of Four Strains Bacteria with Potential to Biodegrade Polyethylene and Polypropylene from Mangrove. Microorganisms.

[147] Ullah Jamil S.U., Zada S., Khan I., Sajjad W., Rafiq M., Shah A.A., Hasan F. (2017). Biodegradation of polyethylene by bacterial strains isolated from Kashmir Cave, Buner, Pakistan. J. Cave Karst Stud..

[148] Bandopadhyay S., English M., Anunciado M.B., Starrett M., Hu J., Liquet y González J.E., Hayes D.G., Schaeffer S.M., DeBruyn J.M. (2023). Organic and inorganic nitrogen amendments reduce biodegradation of biodegradable plastic mulch films. Soil.

[149] Sharma K., Sharma M., Thakur N., Ullah H., Hassan S.H.A., Zheng Y., Li X., Sakran M., Salama E.-S.S. (2025). Enhancement of environmental microplastics (MPs) degradation via bacteria under stress conditions: Key enzymes, pathways, and mechanisms. World J. Microbiol. Biotechnol..

[150] Bogdanov K.I., Kostina N.V., Plakunov V.K., Zhurina M.V. (2022). Effect of Humic Acids on Biofilm Formation on Polyethylene Surface and Its Biodegradation by Soil Bacteria. Eurasian Soil Sci..

[151] Nowak B., Rusinowski S., Chmielnicki B., Kamińska-Bach G., Bortel K. (2016). Degradation of PVC/rPLA Thick Films in Soil Burial Experiment. IOP Conf. Ser. Earth Environ. Sci..

[152] Giyahchi M., Moghimi H. (2025). Sustainable solution for microplastic removal: Sequential biodegradation and detoxification of polyethylene terephthalate microplastics by two natural microbial consortia. Ecotoxicol. Environ. Saf..

[153] Szczyrba E., Pokynbroda T., Koretska N., Gąszczak A. (2024). Isolation and characterization of new bacterial strains degrading low-density polyethylene. Chem. Process Eng. New Front..

[154] Kawai F. (2015). Biodegradation of Polyethylene Glycols and Polyethoxylated Surfactants. Polyethers and Polyethylene Glycol: Characterization, Properties, and Applications.

[155] Liu X., Wang X., Wang R., Guo S., Ahmad S., Song Y., Gao P., Chen J., Liu C., Ding N. (2023). Effects comparison between the secondary nanoplastics released from biodegradable and conventional plastics on the transfer of antibiotic resistance genes between bacteria. Environ. Pollut..

[156] Lu H., Diaz D.J., Czarnecki N.J., Zhu C., Kim W., Shroff R., Acosta D.J., Alexander B.R., Cole H.O., Zhang Y. (2022). Machine learning-aided engineering of hydrolases for PET depolymerization. Nature.

