# Supplementary material for: Microbial Biodegradation of Plastics: Methods for Assessing Degradation Mechanisms and Optimization Strategies
Source: Microorganisms. 2026 Oct 13;14(10):2335. doi: 10.3390/microorganisms14102335 (PMC13707969; doi:10.3390/microorganisms14102335)
Supplement: Supplementary file 1 [file microorganisms-14-02335-s001.zip › microorganisms-4553754-supplementary.pdf]

Supplementary Materials:

Microbial Biodegradation of Plastics: Methods for assessing, Degradation Mechanisms, and Optimization Strategies

Yuming Qiao [1,2], Yunqiao Wang [3], Guoqiang Han [1,2], Jingzhen Zhang [1,2], Zhiyuan Yao [1,2], Bin He [1,2], Pengfei Hu [1,2,*] and Li Jiang [1,2,*]

[1] *Institute of One Health Science, School of Civil Engineering and Future Cities, Ningbo University, Ningbo 315211, China*

[2] *International Science and Technology Cooperation Base for Coastal Agricultural Water Resilience and One Health of Zhejiang Province, Ningbo 315211, China*

[3] *Sichuan WEIPU Testing Technology Co., Ltd., Chengdu 610100, China*

** Correspondence: hupengfei@nbu.edu.cn (P.H.); jiangli@nbu.edu.cn (L.J.)*

Text S1: Literature searching and filtering description.

Literature retrieval for this review was primarily performed via the Scopus database, with a customized search time frame spanning from 2000 to 2026. Scopus is a comprehensive, multidisciplinary, and authoritative abstract and citation database covering extensive peer-reviewed journals across environmental and biological disciplines, which guarantees broad coverage and high representativeness of studies on plastic microbial degradation. To further expand the retrieval scope, compensate for potential database limitations, and avoid missing relevant literature, the Web of Science Core Collection was adopted as a supplementary database with a search time range covering 2000-2026 for complementary retrieval and cross-validation. The final literature search was conducted on March 7, 2026. The search was conducted using a combination of core keywords, including “plastic”, “microbial degradation”, “microbial decomposition”, “microbial breakdown”, “mechanism”, and “efficiency”. A multi-stage systematic screening process was adopted to select eligible literature for this review. Importantly, all literature screening work is implemented based on objective and standardized academic criteria to prevent subjective exclusion of valuable studies. To assess the reliability and rigor of all included studies, the inclusion and exclusion criteria based on three aspects: literature type, research subject, and research content was proposed (Table S1). And the overall literature screening workflow and statistical results are visually summarized in Figure 1.

Literature retrieved from Scopus (2000-2026) and Web of Science Core Collection (2000-2026) was imported into Literature management software (Zotero Ver7.0.32). Duplicate records with identical titles, authors, and publication years were systematically removed. For literature with similar titles but different publication information or experimental systems, manual discrimination and reservation were performed to ensure the uniqueness and effectiveness of all included studies. The initial search returned 3614 articles, from which 1869 duplicates were removed. Therefore, the remaining 1745 articles were screened and excluded 548 review, short communications, commentaries,

letters, conference abstracts and book chapters. The titles and abstracts of the remaining 1197 studies were independently screened with the inclusion and exclusion criteria (Table 1). After careful reading of titles and abstracts, the 785 articles that are inconsistent with the topic were filtered out. And 45 articles for which the original text could not be retrieved were excluded. The remaining 367 reports were further evaluated to assessing the quality, and 125 records were excluded. And the 86 of the remaining 242 studies were excluded because they lacked detailed degradation mechanisms or performance data. Disagreements were resolved through discussion or by consultation. Finally, 156 articles were selected for full text review and included in the study. A total of 156 articles were selected for full text review and inclusion.

Table S1. The inclusion and exclusion criteria for publications

**1. Literature type**
**Inclusion criteria**: Peer-reviewed original research articles that focus on the microbial biodegradation of plastics, including relevant degradation mechanism exploration, degradation assessment methods, and biodegradation optimization strategies. Only formally published, complete academic works with available full-text data and detailed experimental or analytical results were included.   **Exclusion criteria**: Review, short communications, commentaries, letters, conference abstracts, book chapters without complete original experimental data. In addition, studies that only provided simple descriptions, qualitative summaries, or irrelevant topic discussions without valid biodegradation-related results were excluded.
**2. Research Subject**
**Inclusion criteria**: Studies focusing on microbial decomposition, breakdown, and biodegradation behaviors of plastic materials. The eligible publications must investigate the interactions between microorganisms (including bacteria, fungi, and microbial consortia) and plastic substrates, covering plastic degradation characteristics, microbial metabolic responses, and corresponding environmental and functional performances during the biodegradation process.   **Exclusion criteria**: Pure physical or chemical degradation studies of plastics without microbial participation, studies focusing solely on plastic pollution detection, environmental concentration monitoring, or physical/chemical modification of plastics that do not involve microbial biodegradation processes. Studies unrelated to plastic microbial degradation mechanisms, assessment methods, or optimization strategies were also excluded.
**3. Research Content**
**Inclusion criteria**:   (1) Studies that systematically explore microbial biodegradation pathways, core degradation mechanisms, and key influencing factors affecting plastic biodegradation performance.   (2) Studies that construct or adopt scientific assessment methods to evaluate plastic biodegradation efficiency, degradation degree, and corresponding microbial functional characteristics.   (3) Studies that propose or verify optimization strategies for improving plastic microbial degradation performance, including microbial strain screening, culture condition optimization, pretreatment modification, and synergistic degradation systems.   (4) Studies that provide valid experimental data, plastic characterization results, microbial activity data, or quantitative biodegradation performance indicators to support mechanism analysis and strategy evaluation.   **Exclusion criteria:**   Studies lacking detailed effective assessment data. Papers with insufficient experimental information, or only macroscopic phenomenon descriptions were excluded.

Furthermore, studies failing to meet the following quality requirements were strictly excluded:<br>(1) Defective experimental design: studies without clearly defined experimental groups, blank or control groups.<br>(2) Incomplete core data: studies missing key quantitative indicators, such as plastic biodegradation rate, plastic structural characterization data, and functional performance results.<br>(3) unreasonable research methodology: studies adopting non-standard, unscientific detection and assessment methods for plastic biodegradation evaluation.<br>(4) unreliable research results: studies whose core conclusions cannot be fully verified and supported by valid experimental data and statistical analysis.

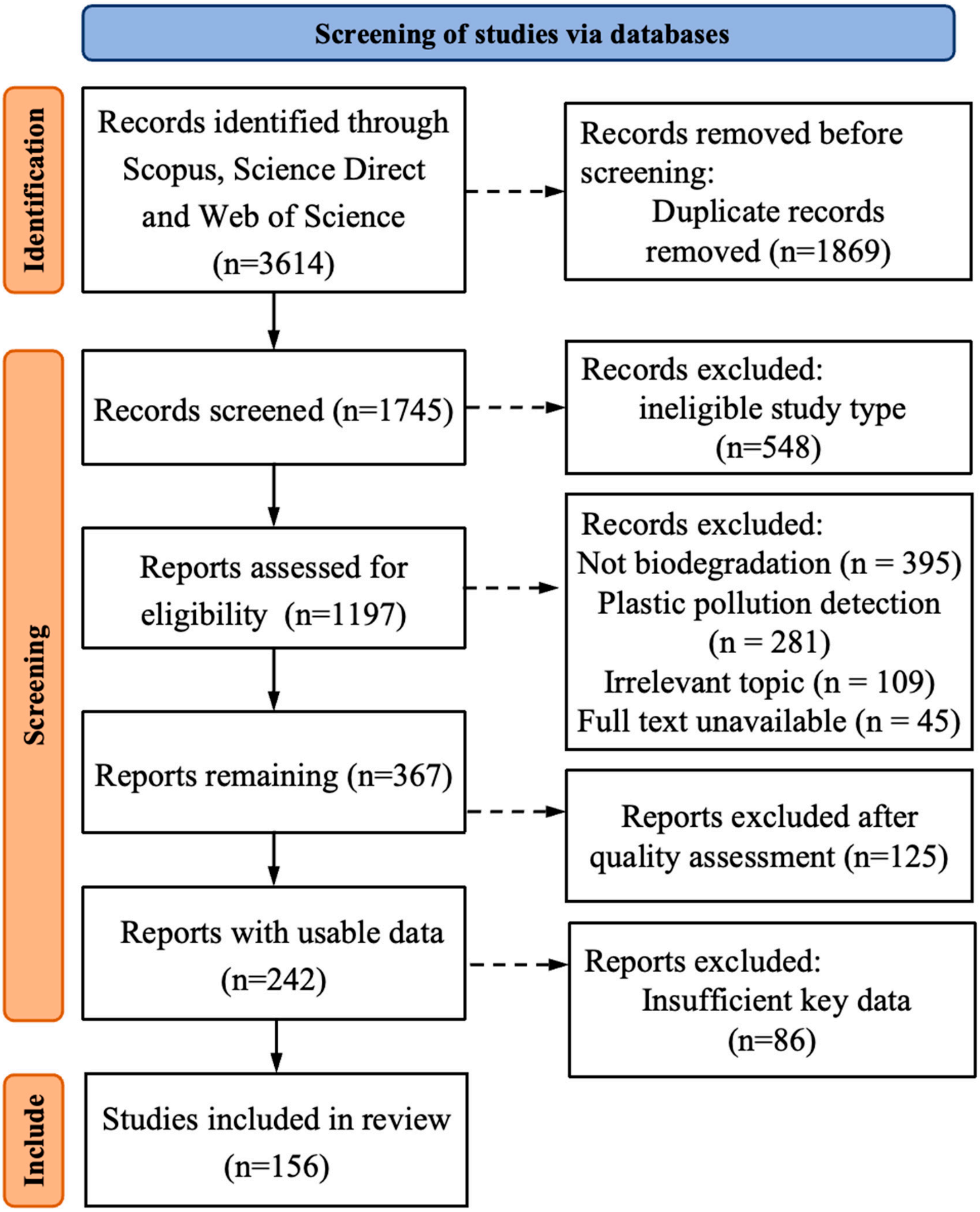

Figure S1: The flow diagram for literatures filtering.
